# Introducing Mr. Three: Attention, Perception, and Meaning Selection in the Acquisition of Number and Color Words

**DOI:** 10.1162/opmi_a_00163

**Published:** 2024-09-15

**Authors:** Katharine A. Tillman, Katie Wagner, David Barner

**Affiliations:** Department of Psychology, University of California, San Diego; Department of Psychology, The University of Texas at Austin

**Keywords:** number cognition, number words, proper nouns, cognitive development, color words

## Abstract

Children learn their first number words gradually over the course of many months, which is surprising given their ability to discriminate small numerosities. One potential explanation for this is that children are sensitive to the numerical features of stimuli, but don’t consider exact cardinality as a primary hypothesis for novel word meanings. To test this, we trained 144 children on a number word they hadn’t yet learned, and contrasted this with a condition in which they were merely required to attend to number to identify the word’s referent, without encoding number as its meaning. In the first condition, children were trained to find a “giraffe with three spots.” In the second condition, children were instead trained to find “Mr. Three”, which also named a giraffe with three spots. In both conditions, children had to attend to number to identify the target giraffe, but, because proper nouns refer to individuals rather than their properties, the second condition did not require children to encode number as the meaning of the expression. We found that children were significantly better at identifying the giraffe when it had been labeled with the proper noun than with the number word. This finding contrasted with a second experiment involving color words, in which children (*n* = 56) were equally successful with a proper noun (“Mr. Purple”) and an adjective (“the giraffe with purple spots”). Together, these findings suggest that, for number, but not for color, children’s difficulty acquiring new words cannot be solely attributed to problems with attention or perception, but instead may be due to difficulty selecting the correct meaning from their hypothesis space for learning unknown words.

## INTRODUCTION

Number word learning poses a difficult challenge to young children. Even after they learn to recite a partial count list (“*one*, *two*, *three* …”) at around age 2, it takes children several more years to demonstrate an understanding of how counting represents number (e.g., Carey & Barner, 2019; Davidson et al., 2012; Fuson, 2012; Le Corre & Carey, 2007; Le Corre et al., 2006; Mix et al., 2002; Sarnecka & Lee, 2009; Siegler, 1991; Wynn, 1990, 1992). Prior to learning how the counting procedure is used to generate precise cardinalities, children pass through a series of well-documented stages in which they acquire exact meanings for their first three or four number words in sequence, and learn to give appropriate amounts when asked to do so in a task that is known as Give-a-Number or Give-N (Marchand et al., 2022; Schaeffer et al., 1974; Wynn, 1990, 1992). During this “subset-knower” period, children transition from being “non-knowers” (who have meanings for no number words), to “one-knowers” (who have an exact meaning for *one*, and give one when asked for one, but not for larger numbers), to “two-knowers” (who have exact meanings for *one* and *two*), to “three-knowers”, and then, sometimes, “four-knowers,” before eventually becoming “CP-knowers” who realize that counting while pointing at objects can be used to identify the cardinality of sets (see Carey, 2009 for a review). Interestingly, there are long delays between the “subset-knower” stages. For example, it takes roughly half a year between acquiring an exact meaning for “one” and an exact meaning for “two,” and there is a similar delay between acquiring “two” and “three” (e.g., Le Corre & Carey, 2007; Sarnecka & Lee, 2009; Wynn, 1990, 1992). Why is it that a child who has already figured out how to give sets of exactly 1 and 2, and can recite the count-list to 10 or higher, nonetheless struggles for months before being able to produce a set of exactly 3 in response to requests for “three”?

Multiple factors are known to impact the rate at which different children acquire the meanings of number words. These include the availability of informative linguistic cues like number morphology (Almoammer et al., 2013; Barner, Chow, et al., 2009; Barner, Libenson, et al., 2009; Bloom & Wynn, 1997; Le Corre et al., 2016; Li et al., 2009; Marušič et al., 2016), the frequency and quality of caregiver input (Eason et al., 2021; Gunderson & Levine, 2011; Levine et al., 2010; Mix et al., 2012; Shneidman et al., 2013), IQ, counting ability, and understanding of relative quantity (Geary et al., 2019), as well as SES and multilingualism (Sarnecka et al., 2021; Wagner et al., 2015). However, amidst these factors that explain individual differences, previous studies have also suggested that the problem of learning number words might be uniquely challenging to children, relative to other instances of word learning, and have described at least three different ways that this might be so.

First, some studies have argued that number word learning may be uniquely difficult because of the perceptual noise associated with non-verbal representations of number. Although some studies demonstrate that very young preverbal infants robustly represent small sets—including comparisons of 1 vs. 2 and 2 vs. 3 (e.g., Feigenson et al., 2002; Feigenson & Carey, 2003)—other work emphasizes that their ability to do so nevertheless differs as a function of the ratio between the quantities being compared, even for relatively small arrays of 2 or 3 objects (Cantrell et al., 2015; Cantrell & Smith, 2013; Schneider et al., 2022). Such results could reflect noise in representing individual objects in visual working memory (see Brady et al., 2024), or it could be due to noise associated with the use of the “approximate number system” or ANS (Dehaene, 1997, 2011; Feigenson et al., 2004; Gallistel, 1990; Meck & Church, 1983), which on some accounts is responsible for representing quantities in the small number range (e.g., Cheyette & Piantadosi, 2020). Representations in the ANS system are characterized by Weber’s law, such that representations become increasingly noisy and approximate as sets grow larger (e.g., Halberda & Feigenson, 2008). Compatible with this *perceptual discrimination hypothesis*, previous studies find that subset-knowers are far from perfect when matching sets even when they are very small (e.g., sets of 2–3) and within their knower-level (Mix, 1999; Negen & Sarnecka, 2009; Schneider et al., 2022). Also, several studies report that subset-knowers exhibit noisy knowledge of number words when they first acquire them (Barner & Bachrach, 2010; Gunderson et al., 2015; O’Rear et al., 2020; Wagner et al., 2019). Together, these studies suggest that perceptual representations of both large and small set sizes may be noisy, which might in turn pose a problem for child learners of exact number words.

A second factor that might impact the availability of exact number meanings in early learning is the relative salience of number to children. On this view, it’s possible that children can perceive differences between small numbers reliably enough to support learning, but that number is simply a less salient dimension of experience than competing features of scenes, such as the shapes of objects (Biederman, 1987; Booth & Waxman, 2008; Landau et al., 1988; Marr & Nishihara, 1978; Strauss & Cohen, 1980; Wilcox, 1999; Woodward, 2000). As noted by Bloom and Wynn (1997), number may be less salient to children because it is a property of sets, whereas many early-learned words label either individual things (e.g., balls, cups), or properties of these things (e.g., shape, size, color, texture). Against this, some studies have argued that number is very perceptually salient, and spontaneously encoded during perception of scenes (e.g., Hannula & Lehtinen, 2005). However, although some children spontaneously focus on number, others do not, and benefit from training (Hannula-Sormunen et al., 2020; see also Goldstein et al., 2016, for possible sources of training in naturalistic conditions). Interestingly, those children who do spontaneously attend to number are faster to learn number words (Savelkouls et al., 2020), compatible with the possibility that the salience of number may limit learning in some children. If children fail to attend to number despite being able to discriminate between different quantities, they may also fail to identify number as a potential referent for number words, a possibility we refer to as the *perceptual salience hypothesis*.

Perceptual properties like discriminability or salience may not alone explain the difficulties children have learning words like *one* and *two*. Even if number is salient to children and they are able to perceive differences between quantities, they may fail to identify the meanings of specific number words because many alternative types of numerical meanings are possible. Just in English, for instance, a set of 3 can be linguistically quantified in many different ways, including via words like *some*, *all*, *few*, *several*, or via the use of plural nouns, all of which express number in different ways. For example, a set of exactly 3 giraffes could be described in English as “some giraffes,” “a few giraffes,” or just “giraffes,” among other possibilities. A number of previous studies have argued that, initially, children may entertain such possibilities as meanings for number words like *three*, and may interpret unfamiliar numbers as plural forms (for discussion, see Barner et al., 2012; Carey, 2004; Clark & Nikitina, 2009; Feiman et al., 2019). Furthermore, children learning English may also consider meanings that are found only in other languages. Some languages, such as Slovenian, include singular, dual, and trial forms to denote sets of exactly one, two, or three (Almoammer et al., 2013; Marušič et al., 2016, 2021), and others have plural forms that are used only for sets of 3–5, or that are restricted to sets larger than 5, or specifically sets of 5–10, depending on the specific language (e.g., Corbett, 2000), creating a very wide range of possible meanings. Given these facts, it is possible that even if number is salient to children and they can reliably discriminate quantities, they may be unsure *which* numerical meaning to assign to a novel number word, since meanings like “several”, “paucal”, or “plural” may be given equal or greater consideration than meanings like “exactly two” or “exactly three”. We call this the *meaning selection hypothesis*.

Here we ask to what extent the perceptual discrimination, perceptual salience, and meaning selection hypotheses might explain why children are so slow to advance from one subset-knower stage to the next and whether the problem of number word learning is unique in the challenge it poses to children. To do so, we exploited a subtle yet important difference in the way that words encode conceptual and perceptual content. Whereas some words, like adjectives, encode properties like number, color, size, and shape directly, other words depend on this information without necessarily encoding it as part of their core meanings. For example, whereas it might be important to notice that Big Bird is big when learning his name (e.g., to tell him apart from other, smaller, birds), it isn’t strictly necessary to know that the word “big” encodes size. In general, proper nouns don’t express properties like size and shape as their lexical meanings, even if these properties are useful for identifying their referents. This means that to learn Big Bird’s name, all that’s required is that size is salient, and that the learner is able to tell apart small and large sizes. In contrast, to learn the meaning of the adjective “big”, not only are salience and discriminability necessary, but it is also critical to know that “big” directly encodes size, in order to permit generalization of the expression to novel instances. So, the learner must notice size, tell small and large sizes apart, and then infer that size is what’s encoded by the adjective “big” from among a range of other competing hypotheses. Given this distinction, we reasoned that if number word learning is hard due to either perceptual salience or perceptual discrimination, then this should equally impact children’s learning of both number words and the proper names of characters who are distinguished by numerical properties. On the other hand, if number is salient and discriminable to children but exact meanings are difficult to select from a range of alternative candidate numerical meanings, then children may readily use number to identify the referents of proper nouns, but fail to identify exact cardinality as the meaning of unknown number words.

Compatible with this intuition, previous studies report that children are able to use color properties to differentiate the referents of proper nouns, even before they have encoded these same properties as the meanings of corresponding color adjectives. By the time children are one year old, they recognize that words introduced in contexts like “This is *Dax*” are proper nouns that indicate unique individuals (Bélanger & Hall, 2006; Hall et al., 2003; Katz et al., 1974; LaTourrette & Waxman, 2020), and by as early as 21 months, they know that words introduced in contexts like “This one is *blickish*” are adjectives that indicate properties of items (Booth & Waxman, 2003; Mintz, 2005; Mintz & Gleitman, 2002; Waxman & Booth, 2001; Waxman & Markow, 1998). By age 3, children can use this syntactic difference to assign a different interpretation to the same word, like “red”. For example, in a study by Hall et al. (2003), children who knew the meaning of the adjective *red* were told a story about a red character who was described either “Mr. Red” or “the red one,” and watched as the character was covered in green paint. When shown this now green character next to a similar red character, children who were asked to identify “Mr. Red,” chose the original (now green) character, whereas children who were asked to point at “the red one,” were more likely to choose the new red individual. Critical to the current investigation, in another study, when 2-year-olds who had not yet acquired meanings for any basic color adjectives (e.g., *red*, *green*, *yellow*) were shown two toys that differed only in color, they easily learned that the red one (and not, e.g., the tan one) was named *Emily*, even though they couldn’t correctly identify the correct referent of *red* from a set of basic colors (Soja, 1994). This suggests that although color is salient and discriminable to young children, they may struggle to encode hue when learning the meanings of color words (see Wagner et al., 2013, for evidence that young children consider multiple types of meanings for color words, including hue, value, and chroma, e.g., with some labeling achromatic colors such as white, black, and brown together under one label).

These previous studies of color word learning raise the possibility that dimensions of experience like color and number may be difficult to encode linguistically despite being perceptually available, compatible with the meaning selection hypothesis. However, this question has not been directly tested in either case. First, in the case of number, no experiments analogous to those conducted by Soja (1994) have been performed, leaving open whether number is also available to children as a salient dimension that might support the learning of proper nouns. Second, even in the case of color words, it is not clear whether meaning selection is indeed especially difficult. Although Soja reported that children in her study had not yet acquired color word meanings, she did not directly compare whether learning a proper noun label (e.g., “Emily” or “Mr. Red”) for a red object is more or less challenging than learning the adjective “red” under comparable conditions, since in her study no color adjective training was conducted. This leaves open the possibility that if children had been trained on color words, they also would have succeeded in her study. Also, studies subsequent to Soja’s have found that children actually begin to acquire color word meanings much earlier than previously thought, and exhibit evidence of preliminary knowledge of color words by around 18 months of age (Forbes & Plunkett, 2019; Wagner et al., 2013, 2018). Although these meanings are not yet adult-like (e.g., some children conflate pink, purple, and red), they may be sufficient to differentiate some of the colors used in Soja’s proper noun condition (i.e., tan and red). Thus, children in Soja’s study may not have actually lacked meanings of color words. Given this, it remains unclear whether learning color words is indeed more challenging to children than using color properties to learn proper nouns, and thus whether meaning selection or perceptual salience pose challenges to children, whether in the case of color, or other cases like number.

Here, we conducted two studies to investigate the role of perceptual discrimination, perceptual salience, and meaning selection in the acquisition of number words and color words. First, in Study 1, we used methods adapted from Soja’s studies of color word learning, and attempted to teach 1- and 2-knowers either a proper noun, e.g., “Mr. Three,” or a number word in adjectival form, e.g., “giraffe with *three* spots.” After training, we assessed children’s ability to (1) re-identify the target giraffe, (2) transfer their learning of three to identify a different 3-spotted exemplar, and (3) to give precisely three objects in the classic Give-a-Number task. Using this method, we asked whether children who haven’t yet learned the meanings of small number words can nevertheless use numerical properties to learn proper nouns. If so, this might suggest that the bottleneck children face when moving from one knower level to the next is due to the problem of identifying exact cardinality as relevant to the word’s meaning, rather than a limit due to attention or perceptual discrimination. However, if children’s difficulty with learning number words is due to a failure of attention or discrimination, then they should find it equally hard to learn proper nouns and number adjectives.

In Study 2, we revisited the question of color word learning. As already noted, although previous studies argue that children can use color information to learn names but not color adjectives, this question has not been directly tested by previous studies, since those studies did not attempt to train children on color adjectives. Study 2 therefore used methods similar to those of Study 1 to examine this question in the domain of color. In particular, we tested whether children who did not yet know the word “purple” could be trained to identify “Mr. Purple” vs. “the giraffe with purple spots.” Because of the differences across studies in participant selection criteria, the ages of the children, the content of the words tested, and the details of their methods, we present these as two separate studies, acknowledging that many factors might explain any differences that exist between them. Nevertheless, by including both domains, we hoped to provide a broader portrait of the role that meaning selection, as compared to perceptual salience and discrimination, plays in word learning.

In addition to addressing these primary questions, our study also addressed questions left open by previous studies that trained number word knowledge. In particular, in a study by Huang et al. (2010), 2-knowers who were trained to identify a specific set of “*three* dogs” could later identify other sets of “three dogs”, but did not transfer this learning to identify other sets of three, like three cows. Interestingly, this was the case despite evidence that 3-knowers *could* transfer training on “four” to new exemplars, raising the question of what explained this difference (see Carey et al., 2017, for a replication of the finding for 3-knowers). Also, Huang and colleagues only attempted to train *n*-knowers on *n* + 1, leaving open whether children must learn number words in sequence (e.g., if one must learn “two” before “three”), or if subset-knowers can ever be taught to “skip” a number, in violation of the well-documented sequential acquisition. Here, in addition to testing our primary questions, we also addressed both of these questions using a novel training method that included a transfer phase to test the generalization of learning to novel exemplars, and by training both 1- and 2-knowers on “three”.

## STUDY 1: MR. THREE

### Methods

#### Participants.

We tested 144 2- to 3-year-old children identified as 1-knowers or 2-knowers using the Give-a-Number pre-test described below.^1^ Seventy children participated in the Adjective condition (*M*_age_ = 3.1 years, range = 2.1–4.0 years; 80 girls), and 74 children participated in the Proper Noun condition (*M*_age_ = 2.9 years, range = 2.2–3.8 years).^2^ Children in the Adjective condition included 35 1-knowers and 35 2-knowers. Children in the Proper Noun condition included 41 1-knowers and 33 2-knowers. Children were recruited and tested either in our laboratory at the University of California, San Diego, or in museums and childcare centers in San Diego, CA, Berkeley, CA U.S.A., and in the Comox Valley, British Columbia, Canada. All study procedures were approved by the institutional review board at UC San Diego.

Across the two conditions, an additional 38 children were tested but not included in these analyses^3^ due to failure to complete all required trials (*n* = 23), passing the “giraffe pre-test” described below (*n* = 8), parent interference (*n* = 4), speaking a primary language other than English (*n* = 2), and parent-reported developmental delay (*n* = 1).

#### Procedure.

##### Give-a-Number Pre-Test.

To determine their number knower levels prior to training, children first performed a titrated version of the Give-a-Number task (see Wynn, 1990, 1992) in which they were asked to put different numbers of toys on a plate on each trial. Children identified as 1-knowers or 2-knowers continued in the study.^3^

##### Giraffe Pre-Test.

**Figure 1.**
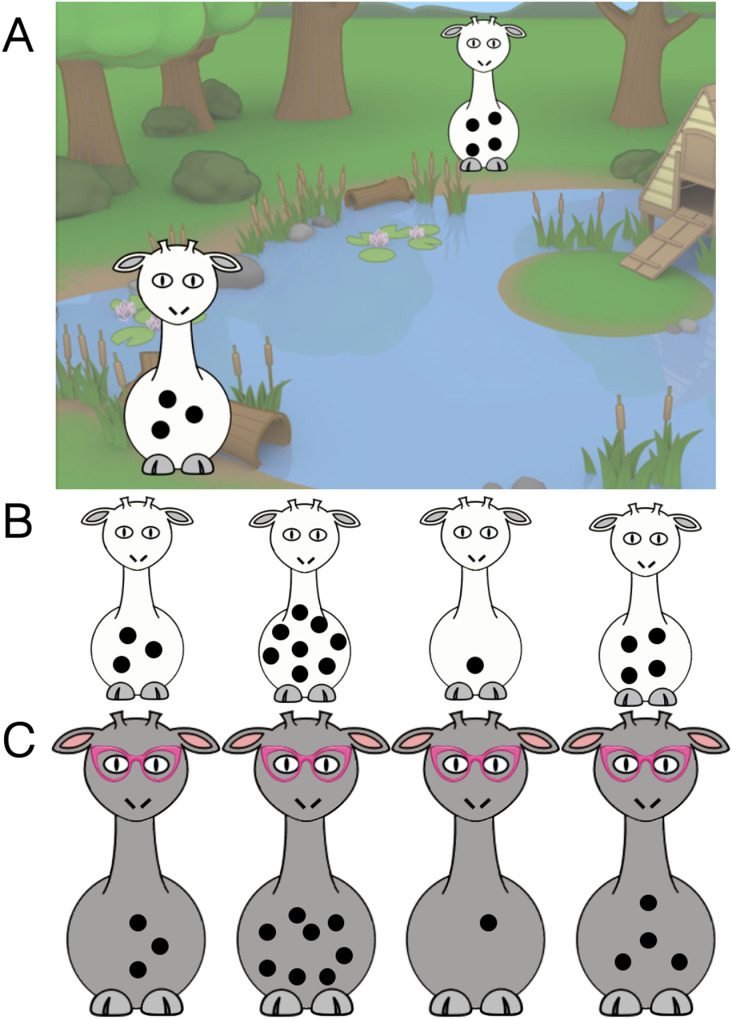
Example stimuli from Study 1. A. Example image from the training story. In the Proper Noun condition, this image was verbally accompanied by, “Next, Mr. Three [point] came to a bench by the road. Look at his tummy! It has spots on it! On the other side of the bench, he saw his friend under a tree. This one [point] is not Mr. Three; it’s his friend …” (see SM for full script). B. Example “line-up” of giraffes used in the Learning test trials and giraffe pre-test. C. Example “line-up” of giraffes used in the Transfer test trials. See Supplementary Materials for additional stimuli; and our Open Science Framework repository for PowerPoint files and scripts.

After the Give-a-Number pre-test, a subset of children (*n* = 89)^4^ were also presented with two “giraffe pre-test” trials, in order to test whether they were able to correctly identify the target 3-spotted giraffe prior to training. On each trial, similar to the Learning test trials described below, the child was presented with a 4-alternative forced choice among cartoon giraffes with 1, 3, 4, or 8 spots (see Figure 1B). Depending on condition, children were asked either to point to the giraffe they thought was “Mr. Three” or “the giraffe with three spots.” No feedback was given. Children who answered both giraffe pre-test questions correctly (*n* = 3 in the Proper Noun condition; *n* = 5 in the Adjective condition) were excluded from all subsequent analyses, as we could not be sure their performance in the test phase was influenced by our training manipulation.

After both pre-test trials were complete, the child was shown the target alone and told that “this one is [Mr. Three/the giraffe with three spots].”

##### Training.

During the training phase, children viewed a slideshow on a computer while the experimenter told a story featuring the 3-spotted target giraffe (see Figure 1A for example stimuli; Supplementary Materials, Section 1, for the complete set of stimuli and story script; and our OSF repository for downloadable scripts and stimulus files). Seven times over the course of the story, children in the Proper Noun condition heard the experimenter label the target giraffe as “Mr. Three” while their attention was drawn to the dots on his stomach (“Look, [child’s name], this is *Mr. Three*. Look at his tummy [point]. It has spots on it”). Children in the Adjective condition heard an identical story in which the target giraffe was instead labeled as “the giraffe with three spots.” In the story, during a walk to his grandmother’s house, the target giraffe encountered three giraffe “friends,” with 1, 4, and 8 spots, once each. These distractors were contrasted with the target, but their associated numbers were not labeled (“This one [point] is *not* Mr. Three, it’s his friend! Look at his friend’s tummy [point]! It has spots too, but it’s different.”). On the final slide, the target giraffe arrived at his grandmother’s house, and her face (but not body or spots) was shown in the window of the house. Importantly, the child never saw the spots on the grandmother giraffe during training. The grandmother giraffe was later used during the Transfer test phase, as discussed below.

##### Learning Test Trials.

Immediately following training, the experimenter said, “That’s the end of the story! Now I need your help. Can you find [Mr. Three/the giraffe with three spots]?” On each of four Learning test trials, the child was presented with a “line-up” of giraffes, including the target and the three distractors from the story (Figure 1B). On each trial, the child was asked to point to Mr. Three/the giraffe with three spots. If the child’s pointing was ambiguous, they were asked to “touch his tummy.”

##### Transfer Test Trials.

Next, the child completed four Transfer test trials in which they were asked to point to either “Mr. Three’s grandma” in the Proper Noun condition or “the grandma with three spots” in the Adjective condition. Again, a lineup of giraffes with 1, 3, 4, and 8 spots was presented (Figure 1C). The configurations of dots differed from those used on Mr. Three and his friends, though the spots were the same size and color.

All children completed the Learning test trials prior to the Transfer test trials. The ordering of the 4 trials within each test phase and the positioning of the 4 giraffes within each trial were counterbalanced across subjects.

##### Give-a-Number Post-Test.

After the Transfer test trials, the experimenter asked children if they wanted to continue. Most children (*n* = 129) said yes, and, to assess whether training impacted their number knower-levels, they completed a second iteration of the Give-a-Number task. The procedure was identical to the Give-a-Number pre-test, except that a different type of toy object was used. Children were not given the Give-a-Number post-test if they indicated that they were tired of playing the game.

##### Coding and Analyses.

Responses in the training task were coded as correct if the child selected the 3-spotted giraffe and incorrect if any other giraffe was selected. All analyses were performed in R, using the *lme4* package for mixed-effects models (Bates et al., 2015). All models included a random intercept for subjects. We performed Wald chi-square tests from type-III analysis-of-variance tables using the *car* package (Fox et al., 2012) to determine whether models including each factor of interest provided a significantly better fit of the data than reduced models.

All data from Studies 1 and 2, along with analysis scripts, are publicly available via the Open Science Framework.

### Results

#### Learning.

If number is salient and discriminable to children, but exact cardinality is not considered as a potential meaning for number words, we should expect children to perform better when using number to learn proper nouns than when learning numerical adjectives. Therefore, to test whether Proper Noun training differed relative to Adjective training, we conducted a mixed-effects logistic regression predicting the likelihood of choosing the correct target, using Knower Level (1-knowers vs. 2-knowers; an ordinal factor) and Condition (Proper Noun vs. Adjective) as predictors, and including an interaction term. We found that the effect of Knower Level significantly improved the fit of this model, *β* = 1.1, *p* = .02; *χ*^2^(1) = 5.2, *p* = 0.02, as did the effect of Condition, *β* = 1.1, *p* = .02, *χ*^2^(1) = 5.3, *p* = .02, but that there was no evidence of an interaction, *β* = 0.21, *p* = .65, *χ*^2^(1) = 0.1, *p* = .75. In other words, 2-knowers were more successful learners than were 1-knowers, and, consistent with the meaning selection hypothesis, children in the Proper Noun condition were more successful learners than those in the Adjective condition.

**Figure 2.**
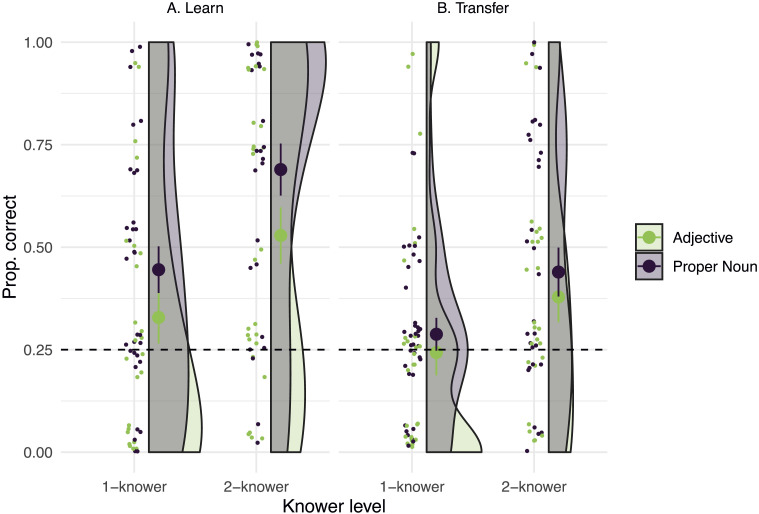
A. Proportion of trials in which children correctly identified either “Mr. Three” in the Proper Noun condition or “the giraffe with three spots” in the Adjective condition, after training. B. Proportion of trials in which children transferred their knowledge to correctly select either “Mr. Three’s grandma” in the Proper Noun condition or “the grandma with three spots” in the Adjective condition. Large dots = group means. Error bars = s.e.m; shaded areas = density of responses at each performance level; dashed line = chance performance.

As shown in Figure 2A, in the Proper Noun condition, 1-knowers correctly identified the target giraffe on 45% (s.e.m. = 6%) of trials, which was significantly greater than chance (i.e., 25% correct; Wilcoxon signed-ranks test, V = 401, *p* < 0.01). Two-knowers in the Proper Noun condition identified the target on 69% (s.e.m = 6%) of trials, also greater than chance (V = 419, *p* < 0.001). Meanwhile, in the Adjective condition, 1-knowers correctly identified the target on 33% of trials (s.e.m = 6%), which was not different from chance (V = 228, *p* = 0.17), while 2-knowers did so on 53% of trials (s.e.m. = 7%), which was greater than chance (V = 334, *p* < 0.001).

Taken together, our findings on the Learning trials show that, as predicted by the meaning selection hypothesis, children in the Proper Noun condition were more likely to learn the identity of “Mr. Three” than children in the Adjective condition were to learn which giraffe was the one “with three spots.” This was true despite the fact that the perceptual demands of the task were equal in both conditions, and children in both conditions were unable to identify the target prior to training. Moreover, only Proper Noun training allowed 1-knowers to identify the 3-spotted target giraffe. One-knowers’ behavior in the Adjective condition was not statistically different from chance.

#### Transfer.

In order to assess whether children’s knowledge of “three” extended beyond the specific exemplars they were trained on in the present study, but within the same basic category (giraffes), we examined performance on the Transfer trials, which involved a new set of giraffes with different spot configurations. A mixed-effects logistic regression with the same effects structure as that used on the Learning trials indicated that neither Condition (*β* = 0.30, *p* = 0.3.; *χ*^2^(1) = 0.91, *p* = 0.3) nor Knower Level (*β* = 0.6, *p* = 0.07; *χ*^2^(1) = 3.3, *p* = 0.07), nor their interaction (*β* = 0.06, *p* = 0.89; *χ*^2^(1) = 0.02, *p* = 0.89) significantly improved the fit of the model predicting success on the Transfer trials relative to a null model. However, as shown in Figure 2B, exploratory *post hoc* analyses found that in the Proper Noun condition, 1-knowers chose “Mr. Three’s grandma” on 29% (s.e.m. = 4%) of trials, which was not different from chance (Wilcoxon signed-rank test, V = 160, *p* = .2), while 2-knowers chose the target grandmother on 44% (s.e.m. = 6%) of trials, which was greater than chance (V = 234, *p* = .002). In the Adjective condition, 1-knowers chose “the grandma with three spots” on 24% of trials (s.e.m. = 6%), which was not different from chance (V = 153, *p* = .7), while 2-knowers chose her on 38% (s.e.m. = 6%) of trials, which was greater than chance (V = 241, *p* = .041). These data provide some preliminary evidence that 2-knowers may show evidence of transfer.

As a follow-up, we next asked whether children who succeeded on Learning trials performed differently on Transfer trials relative to those who performed poorly on Learning trials. To do so, we considered a child to have been successful at Learning if they correctly identified Mr. Three/the giraffe with three spots on at least 3 of 4 test trials (binomial test, *p* = .05). Of the 25 children in the Adjective condition who met this criterion for Learning (36% of the total sample in that condition), 40% (*n* = 10) also succeeded on at least 3 of 4 Transfer trials. Of the 35 children in the Proper Noun condition who met this criterion for Learning (47% of the sample in that condition), 34% (*n* = 12) also succeeded on the Transfer trials. The proportions of learners who transferred their knowledge were not significantly different in the Adjective and Proper Noun conditions (*χ*^2^(1) = 0.03, *p* = .9). In contrast, only 2 children in the Adjective condition and 3 children in the Proper Noun condition who failed to meet the criterion for Learning nevertheless succeeded on the Transfer trials. In both conditions, the proportion of learners who succeeded on the Transfer trials was significantly higher than the proportion of non-learners who did so (both *χ*^2^(1) > 5.3, both *p* < .05).

#### Errors.

**Figure 3.**
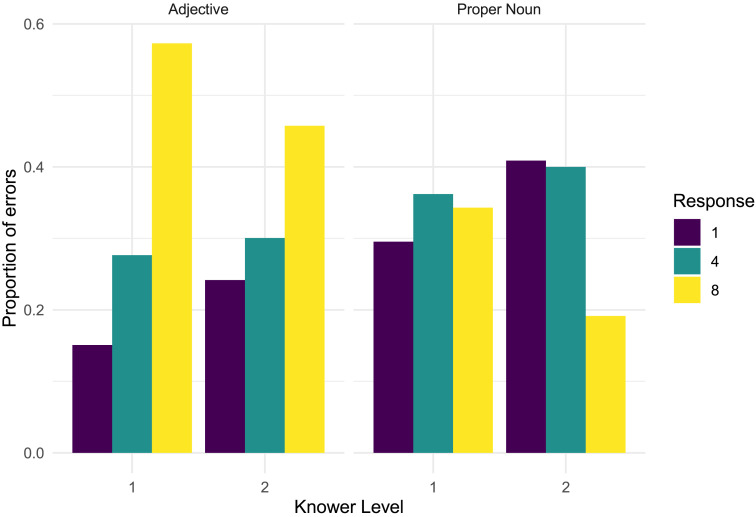
Proportion of x-spotted giraffes chosen in error by children in each knower-level group and condition. One- and 2-knowers in the Adjective condition were less likely to err by choosing 1, which corresponded to a known number word, and most likely to choose 8, while errors were more evenly distributed in the Proper Noun condition.

In addition to the target 3-spotted giraffes, three distractor giraffes featured bellies with sets of 1, 4, and 8 spots. We performed an exploratory analysis of the types of errors children made. The distribution of erroneous responses made by children in each condition and knower-level group is shown in Figure 3.

The three hypotheses about the sources of children’s difficulties with number word learning laid out in the Introduction make different predictions about the types of errors children will make in this task. Specifically, the perceptual discrimination hypothesis predicts that children should be most likely to err by choosing the 4-spotted giraffe, which was most perceptually similar to the target of 3. In contrast, the perceptual salience hypothesis predicts that children’s errors should be randomly distributed across numerosities, given that, on this hypothesis, children are simply not attending to number. Finally, the meaning selection hypothesis also predicts that errors should be random, since they are not considering exact cardinality as a hypothesis, and instead consider hypotheses such as “plural” or “several” which do not differentiate between exact cardinalities.

Contrary to the perceptual discrimination hypothesis, we found that across conditions 1- and 2-knowers were no more likely to err by choosing the 4-spotted giraffe (33% of all errors, see Table S1 in Supplementary Materials for breakdown by condition) than predicted by chance (33%). In fact, children in the Adjective condition were most likely to err by selecting the 8-spotted giraffe, which accounted for over half (52%) of all errors in this condition. This suggests that children’s errors often did not reflect problems with perceptual discrimination - since 3 and 8 are highly discriminable - and are instead compatible with either salience or meaning selection.

There were also differences in the patterns of errors children committed in the Proper Noun and Adjective conditions. Interestingly, children in the Adjective condition, who chose the 4-spotted giraffe on 29% of error trials, were *least* likely to err by choosing the one-spotted giraffe, which was only chosen on only 19% of error trials (significantly fewer than chance, *χ*^2^(1) = 30.4, *p* < 0.001). This was not true of the children in the Proper Noun condition, who selected the one-spotted giraffe on 34% of error trials. This finding is compatible with two interpretations. One is that children in the Adjective condition (but not the Proper Noun condition) used their existing exact knowledge of “one” to exclude the one-spotted giraffe from consideration, consistent with prior work indicating that children can contrast number words they have exact meanings for with those they do not (e.g., Condry & Spelke, 2008; Wynn, 1992). Another possibility is that children used the plural morphology in the test questions to infer that “three” must denote some plural quantity, and not singleton sets. Notably, even if we exclude all errors where children selected 1, we find that children were *less* likely to err by selecting 4 than by selecting the remaining alternative, 8, in the Adjective condition (2-sample test for equality of proportions, *χ*^2^(1) = 47.2, *p* < .001). While this finding is consistent with a general tendency to pick the item with the most dots because it is the most visually interesting, the same pattern was not present in the Proper Noun condition. In the Proper Noun condition, children were *more* likely to err by selecting 4 than 8 (*χ*^2^(1) = 6.8, *p* < 0.01). One possibility is that, in the case of the Adjective condition, but not the Proper Noun condition, some children may have been interpreting “three” to mean “more” or “a lot” (for discussion, see Sullivan et al., 2018).

#### Give-a-Number Post-Test.

Although the prior analyses, as well as previous work (Huang et al., 2010), indicate that some children who are not yet 3-knowers can nonetheless be trained to identify sets of “three” under some circumstances, these findings leave open the question of whether such training changes a child’s knower-level as measured by the Give-a-Number task. Following the training and test phases of the current study, 129 children completed a Give-a-Number post-test (see Table S2 in Supplementary Materials for post-test knower-levels by condition). Strikingly, only 6% of children (*n* = 3 in Proper Noun condition; *n* = 5 in Adjective condition) were classified as 3-knowers or above after completing this study. Seven of these 8 children were classified as 2-knowers at the beginning of the study. Of these 7, all performed better than chance (correctly identifying the target on at least 3 of 4 trials) on the Learning trials, and 2 performed better than chance on the Transfer trials. The majority of children, 67% (*n* = 87), were classified as having the same knower-level at both time points. Of those children whose knower-level changed (*n* = 42), 43% increased (*n* = 18), while 57% decreased (*n* = 24). This level of concordance between pre-test and post-test is nearly identical to the test/retest reliability of the Give-a-Number task, as reported by Marchand et al. (2022) and is compatible with the conclusion that few if any children experienced changes in their knower level in our study, and perhaps also in the previous training study by Huang et al. (2010). In summary, these results suggest that, despite their effectiveness in training identification of sets of three in some children, neither the Proper Noun nor the Adjective training in this study is sufficient to produce new 3-knowers.

### Discussion

The results of Study 1 show that, consistent with the meaning selection hypothesis, children were better able to learn the meaning of a proper noun, “Mr. Three,” than the meaning of a number word presented in a description like, “the giraffe with three spots.” This was the case despite the fact that the target 3-spotted giraffe was the same in both cases and all perceptual demands of the task were identical. If the only limitation children faced in learning the referent of “three” were due to perceptual salience, then we would have expected children to struggle equally in the Proper Noun and Adjective conditions. Similarly, if number were salient and the only problem children had was discriminating numerosities, then we would expect similar performance across conditions. For example, we might expect children to frequently confuse Mr. Three with the 4-spotted giraffe just as they might confuse a “Mr. Eighteen” with a 19-spotted one. However, contrary to this, in the Adjective condition we found that when children made errors they were most likely to pick the giraffe with 8 dots (while errors in the Proper Noun condition were evenly distributed). Given these facts, our results suggest that, in addition to any perceptual difficulties they may face, children also struggle to select exact cardinal meanings from among the many numerical meanings that are available in their hypothesis space. In other words, even when number is salient and discriminable, children do not reliably favor exact cardinality as their first hypothesis when faced with a novel adjective to learn.

Unresolved by this study, however, is whether this difficulty with meaning selection is specific to number words, or if children might demonstrate a distinction between perceiving vs. encoding content in other cases of word learning, too. Indeed, our methods were inspired by the color word learning literature, which shows that even children who do not know any color adjectives can learn proper nouns that designate items that differ only in their color (Soja, 1994). However, no prior studies have pitted color words (i.e., adjectives) and proper nouns against each other in conditions similar to Study 1, to directly test whether one linguistic form is easier for children to learn than the other. In order to test this, in Study 2 we recruited a new sample of children who did not yet know the meaning of the color word “purple” and trained them to identify either “Mr. Purple” or “the giraffe with purple spots” using methods like those of Study 1, but using a set of giraffes whose spots differed in color, rather than number.

If the difficulty children face with selecting the meanings of number words is unique to the domain of number, we would not necessarily expect proper noun training to be easier than adjective learning in the case of color. However, if meaning selection is an equivalent problem for color and number we might expect to see similar differences in learning across these domains. If, on the other hand, the bottleneck children face in acquiring new color words is largely perceptual in nature, we would expect them to have similar degrees of difficulty with proper nouns and adjectives.

## STUDY 2: MR. PURPLE

### Methods

#### Participants.

Fifty-six children (*M*_age_ = 2.3 y, range = 1.6 y–3.2 y; 25 girls) who could not identify the color purple participated in Study 2, including 30 children in the Adjective condition and 26 children in the Proper Noun condition. Note that this sample size was smaller than in Study 1, since here we did not subdivide children into knower levels, given our interest in color, rather than number. Children were recruited from the San Diego, CA (*n* = 51), Berkeley, CA (*n* = 6), and Comox Valley, British Columbia, Canada (*n* = 1) areas. An additional 33 children were tested but excluded from all analyses due to failing to complete all the required trials (*n* = 28), parent interference (*n* = 1), experimenter error (*n* = 2), or insufficient fluency in English (*n* = 2).

#### Procedure.

##### Color Word Pre-Test.

The experimenter laid 11 cards on the table in front of the child. Each card showed an illustration of a fish shaded in with one of 11 basic colors: red, orange, yellow, green, blue, purple, pink, brown, gray, black, or white. On each of four trials, the experimenter asked the child to point to a particular colored fish. The target colors were pink, green, purple, and gray. Only positive feedback was given. Children who correctly selected the purple fish were not included in the study. Note that this is a conservative criterion, as children who know no color words would still be expected to respond correctly by chance 9% of the time (because there were 11 colored fish to choose from). As a result, it is possible that we removed some children who did not know the meaning of the word ‘purple’.

There was no additional “giraffe pre-test” in Study 2 asking children to identify [Mr. Purple/the giraffe with purple spots] prior to training.

##### Training, Learning, and Transfer.

**Figure 4.**
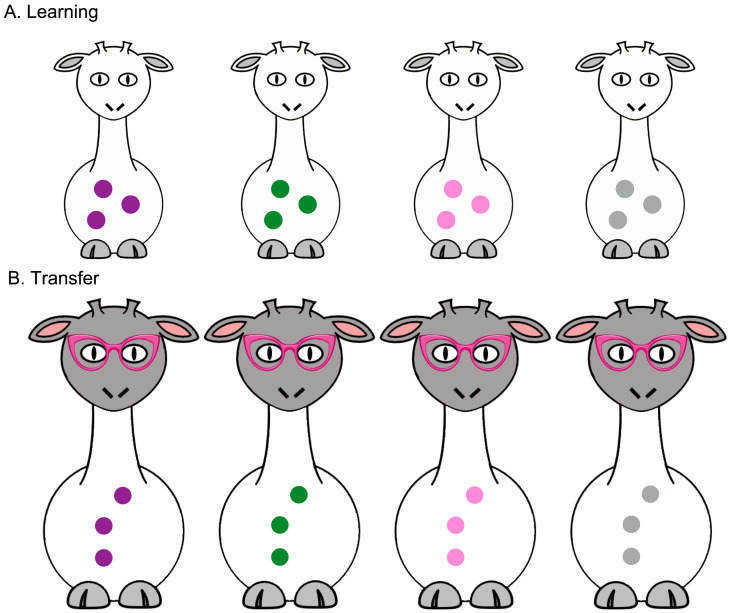
Study 2 test stimuli. A. “Mr Purple” or “the giraffe with purple spots.” B. “Mr. Purple’s grandma” or “the grandma with purple spots.”

Procedures for the Training, Learning, and Transfer trials were identical to those of Study 1 with the following exceptions: As shown in Figure 4, all 4 giraffes had 3 spots on their bellies, and the color (rather than the number) of the spots varied across giraffes: purple, gray, green, or pink. Pink was included as a near distractor to probe whether children made errors in differentiating purple from pink (analogous to the contrast between 3 and 4 in Study 1), while gray and green were included as far distractors (analogous to the numbers 1 and 8 in Study 1). The target giraffe was the one with purple spots. In the Proper Noun condition, children were asked to identify “Mr. Purple” on the Learning trials and “Mr. Purple’s grandma” on the Transfer trials. In the Adjective condition, children were asked to identify “the giraffe with purple spots” on the Learning trials and “the grandma with purple spots” on the Transfer trials.

##### Color Word Post-Test.

Following the completion of the Transfer trials, most children (*n* = 47) repeated the color word comprehension test for a second time. Children who did not complete this color word post-test (*n* = 9) were not excluded from the study.

### Results

#### Color Word Pre-Test.

Children were retained in the sample only if they pointed to an incorrect color when asked to select the *purple* fish during the color word comprehension pretest. For the four words included in the pretest (*purple* plus the 3 distractor colors used in the training), the median percentage correct was 25%, i.e., one correct word. The most frequently known word of the 4 we tested was *green*, which 38% of children in the sample correctly selected. *Pink* was correctly selected by 24% of children in the sample, while *gray* was correctly selected by only 9% of children in the sample.

#### Learning.

**Figure 5.**
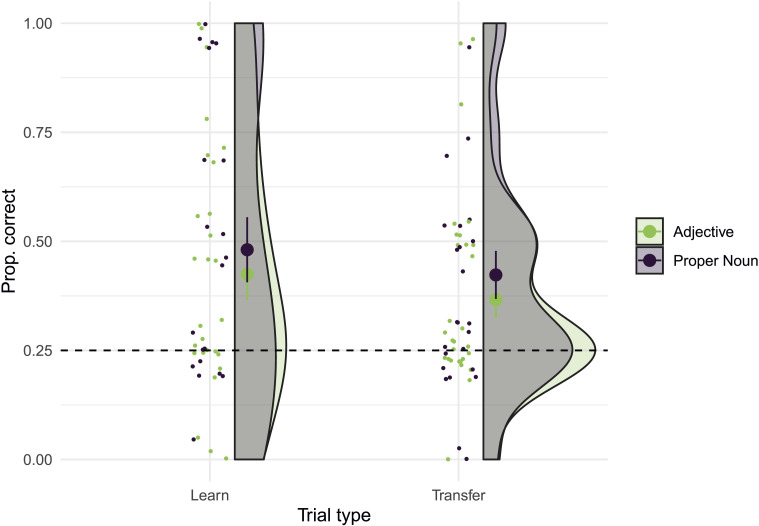
Proportion of Learning trials on which children correctly identified either “Mr. Purple” (Proper Noun) vs. “the giraffe with purple spots” (Adjective) and proportion of Transfer trials children extended their knowledge to correctly select either “Mr. Purple’s grandma” (Proper Noun) or “the grandma with purple spots” (Adjective). Large dots = group means. Error bars = s.e.m; shaded areas = density of responses at each performance level; dashed line = chance performance.

If color is salient and discriminable to children, but children struggle to identify hue from among potential meanings for color words, we should expect children to perform better when using color to learn proper nouns than when learning color adjectives. To test this, we first asked whether children in the Proper Noun condition were more likely to learn the identity of the purple-spotted giraffe than those in the Adjective condition. To do so, we conducted a mixed-effects logistic regression predicting the likelihood of choosing the correct target, using Age (continuous) and Condition (Proper Noun vs. Adjective) as main effects, along with an interaction term and a random effect of Subjects. We found that the effect of Condition did not significantly improve the fit of this model, *β* = −1.5, *p* = .61, *χ*^2^(1) = 0.26, *p* = .61. In other words, children were just as likely to identify the purple-spotted giraffe regardless of whether they had been trained with a proper noun or an adjective. Age also did not significantly improve the fit of the model, *β* = 1.4, *p* = .14, *χ*^2^(1) = 2.1, *p* = .14, which may be a consequence of the relatively restricted age range of the sample and the fact that none of the children could correctly identify purple prior to training. There was no evidence of an interaction, *β* = 0.81, *p* = .51, *χ*^2^(1) = 0.43, *p* = .51. Replicating the finding of Soja (1994), children in the Proper Noun condition correctly identified the target giraffe on 48% (s.e.m. = 6%) of trials (see Figure 4), which was significantly better than chance (i.e., 25%; Wilcoxon signed-ranks test, V = 146, *p* = .004). Additionally, we found that children in the Adjective condition correctly identified the target on 43% of trials (s.e.m = 6%), which was also significantly better than chance (V = 160, *p* = .004). Although mean performance in the Proper Noun condition was slightly higher than for the Adjective condition in Study 2 (see Figure 5), this difference was not statistically significant (Wilcoxon rank sum test, W = 416.5, *p* = .66). Taken together, data from the Learning trials show that children in the Proper Noun and Adjective conditions were equally able to learn the target, suggesting that in this case meaning selection was not a critical barrier to children’s acquisition of color adjectives.

#### Transfer.

A mixed-effects logistic regression with the same effects structure as that used on the Learning trials again indicated that Condition (Proper Noun vs. Adjective) did not significantly improve the fit of the model predicting children’s performance on the Transfer trials, *β* = 2.6, *p* = .13; *χ*^2^(1) = 2.3, *p* = .13, nor did Age, *β* = 0.94, *p* = .10; *χ*^2^(1) = 2.7, *p* = 0.10, and there was no interaction, *β* = −1.0, *p* = .17; *χ*^2^(1) = 1.9, *p* = .17. As shown in Figure 5, we found that, in the Proper Noun condition, children chose “Mr. Purple’s grandma” on 42% (s.e.m. = 6%) of trials. In the Adjective condition, children chose “the grandma with purple spots” on 37% of trials (s.e.m. = 4%). In both conditions, children’s overall performance was greater than chance (Wilcoxon signed ranks tests, both *p*’s < 0.01). In other words, as a group, children displayed evidence of transfer (i.e., they could identify “purple” in a novel context more often than random guessing would predict), and they were equally able to transfer the meaning of ‘purple’ to other exemplars after being trained on the term as an adjective or a proper noun.^5^

As in Study 1, we wondered whether there was a difference in performance on transfer trials for children who did vs. did not show evidence of learning at the individual level. However, due to the very small number of children who succeeded at the criterion level of 3 transfer trials in this study, we were unable to determine whether the effects of learning on transfer differed significantly across the two conditions, or whether children who learned were statistically more likely to succeed at the transfer trials (see Supplementary Materials, Section 4, for details).

#### Errors.

**Figure 6.**
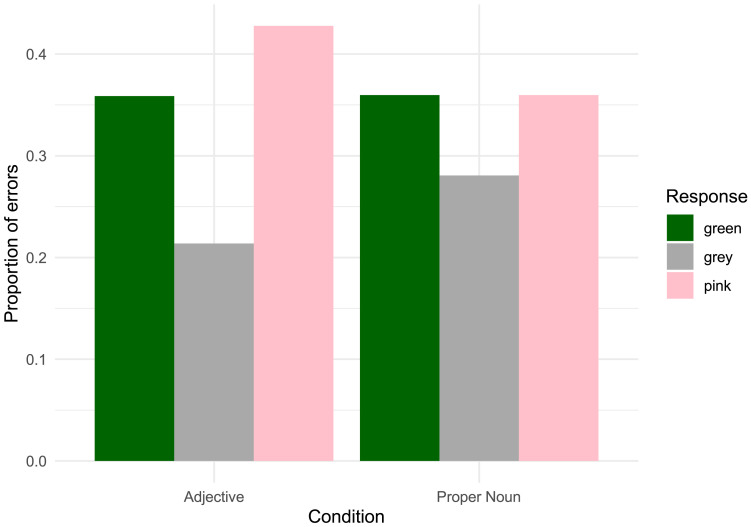
Proportions of distractor giraffes selected in error in Study 2. Children in the Adjective condition were most likely to choose the pink-spotted giraffe, which was most perceptually similar to the target (purple).

In addition to the target purple-spotted giraffes, children also encountered three distractor giraffes with green, pink, and gray spots. The frequency with which each of those colors was chosen on the Learn and Transfer trials is shown in Figure 6. We performed an exploratory analysis of the types of errors children made when they did not select the target. Accounts on which children’s limitation in learning color words is perceptual in nature might predict that they would be most likely to err by selecting the pink giraffe, which was most perceptually similar to the target, in both conditions. On the other hand, the meaning selection hypothesis makes no specific predictions about children’s errors when selecting colors (since on this hypothesis some might focus on chromaticity, others on value, etc.).

We found that children in the Adjective condition were more likely to err by choosing the pink-spotted giraffe (43% of all errors in both Learning and Transfer) than would be predicted by chance (33%; *Χ*^2^(1) = 5.8, *p* = .02). This effect was not significant in the Proper Noun condition, in which children chose the pink-spotted giraffe on 36% of trials, *Χ*^2^(1) = 0.32, *p* = .6. However, the proportions of pink errors in the Proper Noun and Adjective conditions were not significantly different (two-sample test of equality of proportions, *Χ*^2^(1) = 0.96, *p* = .33).

Recall that in the Adjective condition (but not the Proper Noun condition) of Study 1, we found that children were least likely to err by choosing the giraffe with 1 spot, which, unlike the other distractors (and the target) was within their knower-level. We therefore wondered if children in the Adjective condition of Study 2 were also less likely to err by choosing giraffes whose colors they had correctly identified in the color word pretest. In other words, were children who had correctly identified “green” in the pretest less likely to choose “green” when asked for purple during the Learning and Transfer trials? When we examined the errors committed by children in the Adjective condition who knew the distractor colors, we found that 46% of these errors were cases where the child incorrectly selected a color that they already knew the label for, a *higher* percentage than we would expect if children were randomly guessing on error trials (although this difference was not statistically significant, 1-sample proportion test, *Χ*^2^(1) = 2.2, *p* = .13). When we applied the same analysis to children in the Proper Noun condition, we found that 33% of errors made by children who had correctly identified distractor colors reflected colors they had correctly identified in the pretest, which was not different from than chance (one-sample proportion test, *Χ*^2^(1) < 0.001, *p* = 1). Thus we did not have evidence that children in either condition were systematically applying the mutual exclusivity principle, i.e., using their knowledge of the names of the distractor colors to narrow down their choices in the Learning and Transfer trials.

#### Color Word Post-Test.

Following the training and test phases of the current study, 47 children completed the color word comprehension test for a second time. Although all children failed to correctly identify the purple fish prior to participating in the training study, we found that 5 children in the Proper Noun condition (23% of the children in that condition who took the post-test) and 7 children in the Adjective condition (28% of the children who took the post-test) correctly identified the purple fish after the training. These proportions were not significantly different from one another (Wilcoxon rank sum test, W = 260.5, *p* = .69), but only the proportion of children in the Adjective condition was significantly higher than one would predict given random guessing (i.e., 9%, because there were 11 colored fish to choose from; one-sample proportion test, *Χ*^2^(1) = 8.8, *p* = 0.003).

### Discussion

In Study 2, we examined children’s ability to learn and transfer the meaning of ‘purple’ after Adjective or Proper Noun training. In the case of color words, we found success at both learning and transfer for both types of training. Thus, unlike the case of number words in Study 1, we did not find evidence that Proper Noun training was more effective than Adjective training. Also in contrast to Study 1, an error analysis indicated that the perceptual similarity of the target and distractor was a factor in guiding children’s choices in the learning and transfer trials, while the child’s prior knowledge of the names of the distractor colors was not.

These findings contrast with those of prior studies suggesting that color words are more easily learned as proper nouns than adjectives. In particular, Soja (1994) showed that children who failed to identify, e.g., the color red from a set of basic colors could nonetheless be trained to recognize a red object labeled as, e.g., “Emily,” when contrasted with another object that differed only in color. However, whereas that previous study did not attempt to train children to recognize color adjectives and proper names under the same conditions, the present study did, and found no difference between them.

## GENERAL DISCUSSION

Why is it that a child who already has exact meanings for *one* and *two*, and can recite the count list to 10 or above, nonetheless requires several more months to acquire an exact meaning for *three?* Here, we investigated this question by exploring three candidate hypotheses. According to the perceptual discrimination and perceptual salience hypotheses, the bottleneck on early number word learning is due to perceptual factors: either children have a hard time visually discriminating larger arrays from their neighbors in the count-list (e.g., 3 vs 4), relative to smaller arrays (e.g., 1 vs 2), or these numerical features are less salient than other perceptual properties of the scene (e.g., shape or color). In contrast, according to the meaning selection hypothesis, children’s protracted learning of new number words may instead stem from difficulties identifying exact cardinality as the relevant dimension of the stimulus being labeled. For instance, a child might hear a phrase like “the giraffe with three spots” and hypothesize that “three” refers to an alternative numerical meaning such as plurality, rather than exact cardinality.

In our study, we found evidence that supported the meaning selection hypothesis in the case of number word learning, but not color word learning. In Study 1, we found that although some children succeeded, many struggled to identify the referent of an unknown number word, like “three,” when the task required them to encode this information as the meaning of a number word, e.g., “the giraffe with three spots.” In contrast, children performed significantly better at identifying the same giraffe when, instead of needing to learn the number word “three”, they were instead taught the giraffe’s name, i.e., “Mr. Three”. Both forms of learning required children to attend to the number of spots on the tummy of the target giraffe, and to discriminate this number from other quantities that were present on competitor giraffes. However, only the Adjective condition asked children to encode the exact cardinality 3 as the meaning of the number word “three”. This suggests that the process of selecting and encoding a numerical meaning poses a particular challenge to children when engaging in number word learning, above and beyond the problems of noticing number as an important dimension, and beyond the problem of discriminating different quantities.^6^

The finding that children in Study 1 performed worse in the Adjective condition relative to the Proper Noun condition is particularly interesting because they could have simply used the same information to succeed in both conditions: ignoring the problem of figuring out the meaning of “three”, and making their choice based on the perceptual similarity of referents at training and test. The fact that they did not do so in the Adjective condition suggests that when children see their task as learning the meaning of a quantificational expression, they consider hypotheses that otherwise might not be salient, including those that ignore differences in exact cardinality, like “some”, or “a few”. For example, if children encoded the target referent as the “giraffe with *some* spots” during training, this would predict a random selection between giraffes at test (perhaps with a preference for plural sets, as we observed). Such a strategy would be compatible with previous reports, which suggest that children’s earliest meanings for numerals are similar to that of the plural (Barner et al., 2012; Carey, 2004; Clark & Nikitina, 2009).

In contrast, in Study 2 we investigated the case of color, and found that meaning selection did not pose a problem above and beyond the problems of noticing and discriminating between different colors. When children were required to learn a color word like “purple” in order to identify a giraffe, e.g., “the giraffe with purple spots,” they performed just as well as when they were instead taught the animal’s name, e.g., “Mr. Purple”. Thus, unlike in the case of number, we found that once children were able to identify color as relevant and discriminate different colors from one another, the problem of encoding a color as the meaning of an adjective did not pose a significant additional problem to them.

Critically, the results of these two studies do not mean that problems like salience or perceptual discrimination are trivial to children, or that these factors don’t place important constraints on learning. Instead, our data suggest that, at least in the case of number, meaning selection poses an additional, challenging, problem. As evidence that salience and discrimination likely also impact learning, children in both Studies 1 and 2 performed far from perfectly in the Proper Noun conditions, suggesting that they often failed to use number and color cues to discriminate between referents. For example, in Study 1, children identified as 1-knowers only selected the target giraffe 45% of the time in the Proper Noun condition, while 2-knowers did so 69% of the time. Likewise, in Study 2, although children performed significantly better than chance in the Proper Noun condition, children nevertheless failed to correctly identify the target giraffe on 62% of trials. In some cases, such as the Adjective condition of Study 2, errors were compatible with difficulties discriminating perceptual stimuli, as children often chose the giraffe with pink dots when asked to find the one with purple dots. However, in both Study 1 and Study 2, Proper Noun errors were randomly distributed, suggesting that failure in these conditions was often not explained by problems discriminating proximal stimuli (e.g., 3 vs. 4, or purple vs. pink). Instead, these failures in the Proper Noun condition were more likely due to factors like memory, or inattention to the relevant dimension.

As discussed above, while we found that children in Study 1 performed better in the Proper Noun condition than those in the Adjective condition on Learning trials, this was not the case on Transfer trials. There are several candidate explanations for this result. One possibility is that the lack of a Proper Noun advantage on these trials is related to children’s knowledge of the specificity of proper names (Bélanger & Hall, 2006; Hall et al., 2003; Katz et al., 1974; LaTourrette & Waxman, 2020). To the extent that children infer that a proper noun picks out a unique individual (rather than a category, or family of things), one might not expect transfer. Relatedly, a second possibility is that children did not reliably choose “Mr. 3’s grandma” in the proper noun condition simply because they lacked an intuition that a family of giraffes might be perceptually similar, or that the number of spots on a giraffe might be heritable. Compatible with this, one child spontaneously shared her theory that older giraffes should have *more* spots than younger ones. Also, empirical studies of giraffe spots indicate that although the circularity and solidity of giraffe spots are heritable, their number is not (Lee et al., 2018). Further, relevant to Study 2, giraffe spot color changes as giraffes grow older, becoming darker (Berry & Bercovitch, 2012). Because an unintended “intuitive genetics” component of the task could have masked a proper noun advantage we might have otherwise observed in the transfer trials, future research may benefit from avoiding this issue.

Beyond testing the role of meaning selection relative to other factors, our study also makes important contributions to the literature on number word training. First, consistent with prior studies of number-word training (Carey et al., 2017; Huang et al., 2010), exploratory analyses found preliminary evidence that 2-knowers were more likely to learn and transfer the meaning of “three” than were 1-knowers. However, this finding is tempered by the fact that multiple studies find that *n*-knowers often have partial comprehension of the next number in the count list, *n* + 1, and perform non-randomly for this number on a variety of measures (Barner & Bachrach, 2010; O’Rear et al., 2020; Wagner et al., 2019).^7^ Such findings raise the possibility that children in Huang et al. (2010) did not actually change knower levels, but instead deployed existing, partial knowledge, of larger number words, that was sufficient to support labeling in a forced choice task, but not sufficiently robust to qualify for a higher knower level on the Give-a-Number task. Compatible with this, results from our Give-a-Number post-test indicate that children’s knower levels were not substantially impacted by training.

One limitation of our study is that although it shows a clear role for meaning selection in the case of number word learning, it likely underestimates the significance of this problem both for number and for color. Critically, in our studies, we tested children who had already begun the process of learning number and color words.^8^ Consequently, these children had likely already learned that the words “three” and “purple” belong to distinct lexical classes, and therefore that in each case they contrasted in meaning with other members of those classes (for discussion see Shatz et al., 2010). Related to this, although our data suggest that children aren’t especially hindered by meaning selection in the case of color word learning, previous studies suggest that it is likely a major factor for children at earlier stages of learning, when the meanings of fewer competitors are known, and the possible meanings for a word like “purple” are greater in number. For example, as noted in the Introduction, many 2-year-old children entertain meanings that are not present in the lexicons of adult English-speakers, e.g., using a word like “white” to refer to achromatic colors like white, black, and gray (Wagner et al., 2013). Once children have acquired 2-3 color words, hypotheses such as these may be ruled out, greatly narrowing the range of possibilities, and reducing the significance of the meaning selection problem over time.

In conclusion, the current study showed that whereas young children can use numerical cues to identify the referent of a novel proper noun, they often fail to use this same information to acquire exact number word meanings. We also found that, contrary to past reports, this asymmetry is not present for color words. These findings suggest that, when learning number words, children who attend to number and can discriminate different quantities nevertheless struggle to select and encode exact numerical meanings. Future studies should further consider whether the problem of meaning selection is uniquely hard for number, or whether adjectives that represent other dimensions of experience - like time, space, or weight - might pose a similar challenge to young children.

## ACKNOWLEDGMENTS

A portion of the results from Study 1 previously appeared in the *Proceedings of the 40th Annual Meeting of the Cognitive Science Society*. We thank Junyi Chu, Megan Merrick, and numerous other research assistants from the Language and Development Lab at UC San Diego and the Language and Cognitive Development Lab at UC Berkeley for their help with subject recruitment and data collection. We thank Beaufort Children’s Centre, Tigger Too Preschool, Puddleduck Early Learning Society, Forest Circle Child Care, Courtenay Elementary in the Comox Valley, BC; Hearts Leap North, Studio Grow, Habitot, and Peek-a-boo Factory in Berkeley, CA; and University City United Church Preschool and the Fleet Science Center in San Diego, where some of these data were collected. Most importantly, we thank the many children and families who participated in this research.

## FUNDING INFORMATION

Funding for this research was provided by a grant to D.B. from NSF project #1535093.

## AUTHOR CONTRIBUTIONS

KT: Conceptualization, Data curation, Formal analysis, Investigation, Methodology, Visualization, Writing – original draft, Writing – review & editing. KW: Conceptualization, Formal analysis, Investigation, Methodology, Writing – review & editing. DB: Conceptualization, Funding acquisition, Methodology, Supervision, Writing – review & editing.

## DATA AVAILABILITY STATEMENT

Data, analysis code, and stimuli are publicly available via the Open Science Framework: https://osf.io/b2zu5/.

## Notes

^1^ In order to test whether presenting an unknown number word as a proper noun improves children’s learning and identification, we conducted two experiments as part of Study 1, the latter of which was a near-exact replication of the former. All differences between the first and second experiments are noted in the main text. As their methods and the pattern of results they generated were extremely similar, we present combined data from both experiments here, while separate analyses can be found in Supplementary Materials, Section 2, and Figures S1 and S2.^2^ The unequal sample sizes were due to a clerical error resulting in additional children being tested, exceeding our target N. All data were retained.^3^ In the first experiment of Study 1, data from 35 “non-knowers” were also analyzed, as reported in the Supplementary Materials. Non-knowers were not included in the replication experiment or in the results reported in the main text.^4^ This methodological improvement was introduced in the replication experiment, as discussed in the Supplementary Materials.^5^ An anonymous Reviewer noted that the grandmother giraffes had pink glasses, which might be confusing to some children, given that one distractor color was pink (though results indicate that overall children succeeded on transfer). Note that because all grandmothers wore pink glasses, this fact could not explain any bias children had to choose one giraffe over another. However, if children confused pink and purple, it could plausibly lead them to believe that any grandmother with pink glasses was a match.^6^ Note that a deflationary account of our findings for number is that children had more difficulty interpreting the phrase “the giraffe with three spots” because it had more words than “Mr. Three.” While this is possible, we did not find a difference between the Proper Noun and Adjective conditions for Study 2, suggesting that if this was a barrier to children, it was likely modest.^7^ It remains unclear, however, why 2-knowers *also* outperformed 1-knowers in the Proper Noun condition, since success in this condition should not, in principle, require any prior knowledge of any number words.^8^ Although many children failed to correctly identify any of the target words tested in the color word pretest, we intentionally avoided common first color words, such as “red” and “blue,” and thus may have underestimated their knowledge.

## Supplementary Material


