# Supplementary material for: Introducing Mr. Three: Attention, Perception, and Meaning Selection in the Acquisition of Number and Color Words
Source: Open Mind (Camb). 2024 Sep 15;8:1129–52. doi: 10.1162/opmi_a_00163 (PMC11441787; doi:10.1162/opmi_a_00163)
Supplement: Supplementary file 1 [file opmi-08-1129-s001.pdf]

Introducing Mr. Three: Attention, perception, and meaning selection in the acquisition of number and color words

Katharine A. Tillman, Katie Wagner, & David Barner

Supplementary Materials

1. Stimuli for Study 1

Text and illustrations for the giraffe pretest, training, and test phases of Study 1, Experiment 2, are shown below. Complete downloadable scripts and PowerPoint stimulus files are available on our OSF repository.

1.1 Giraffe pretest.

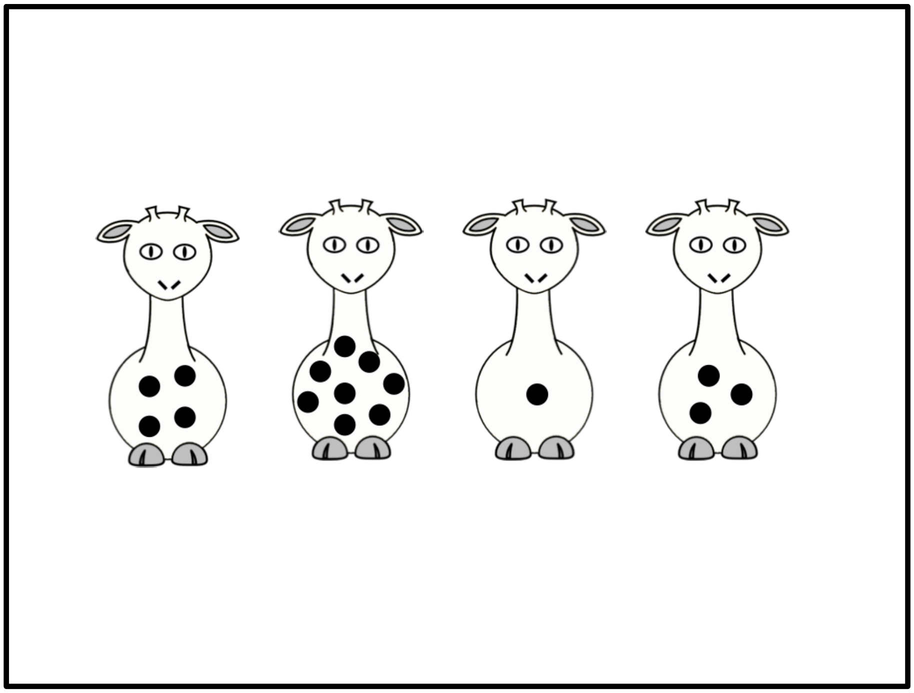

"I'm going to tell you a story about [Mr. Three/a giraffe with 3 spots]. Which one do you think is [Mr. Three/the giraffe with 3 spots]?"

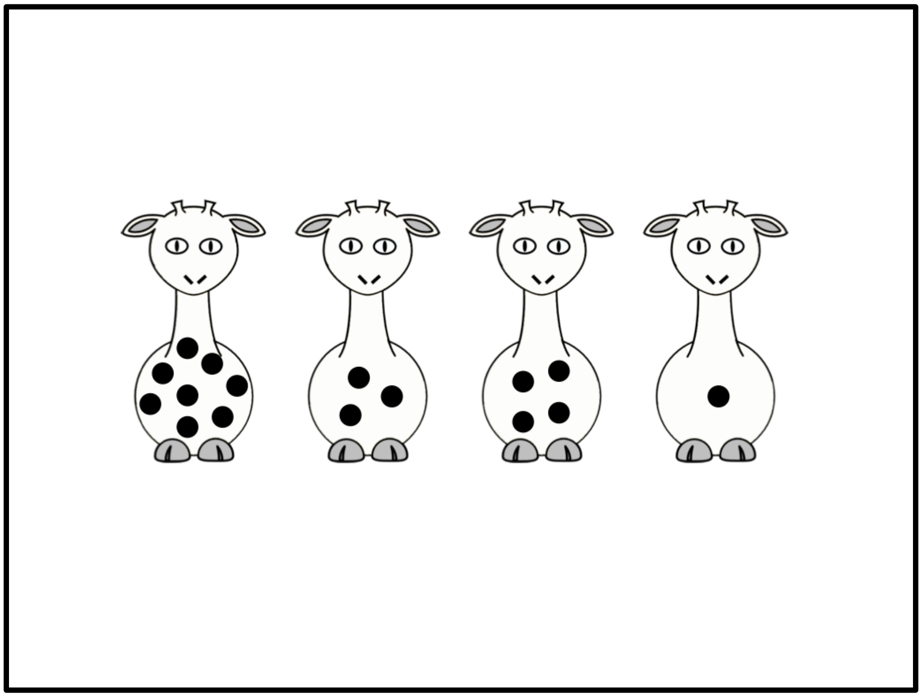

"Can you point to [Mr. Three/the giraffe with three spots]?"

1.2 Training:

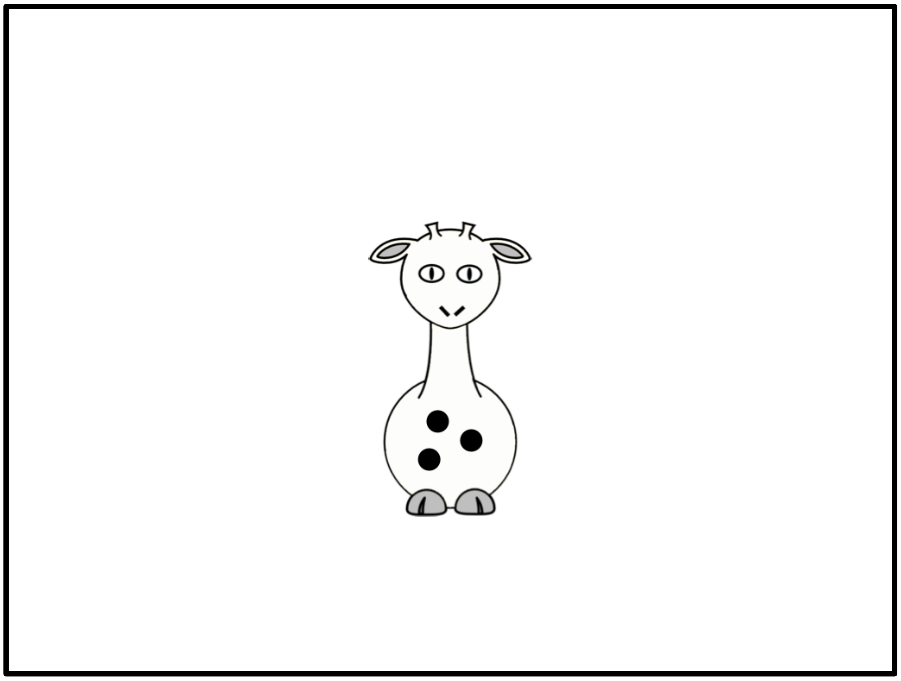

"Look, [child's name], this is [Mr. Three/the giraffe with three spots]. Look at his tummy [point]. It has spots on it. I'm going to tell you a story about [Mr. Three/the giraffe with three spots]?"

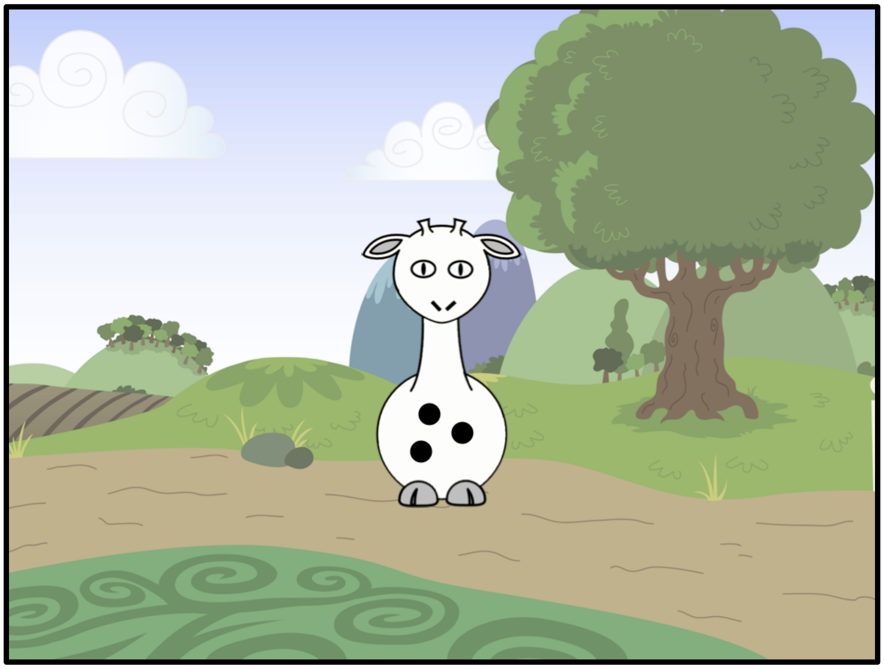

“One day, [Mr. Three/the giraffe with three spots] went for a walk to his grandma’s house. [Mr. Three/the giraffe with three spots] walked down a long road and by a big tree.”

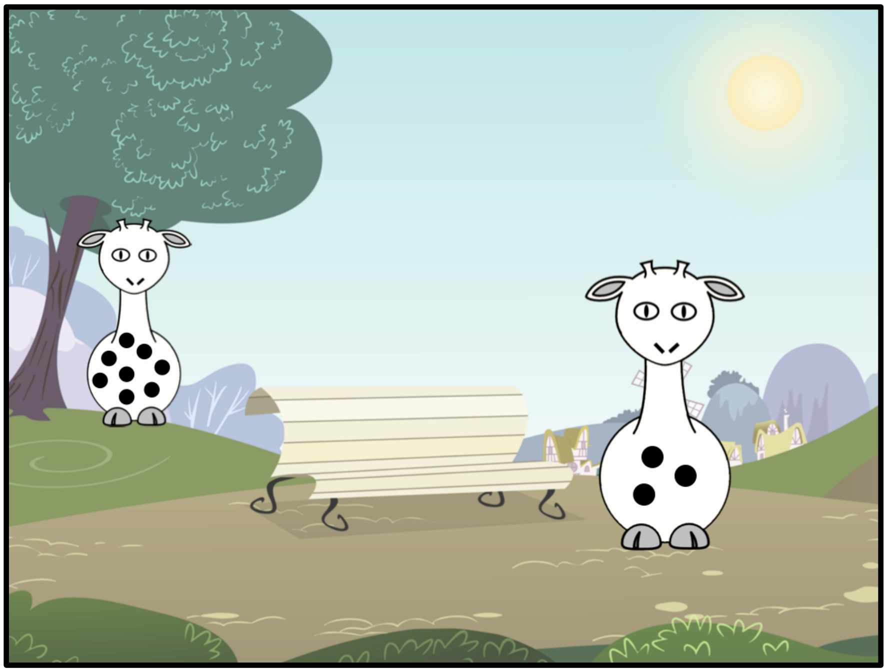

“Then, [Mr. Three/the giraffe with three spots] came to a bench by the road. Behind the bench, he saw his friend under a tree. This one [point] is not [Mr. Three/the giraffe with three spots], it’s his friend. Look at his friend’s tummy [point]! It has spots too, but it’s different. [Mr. Three/the giraffe with three spots] [point] said goodbye to his friend and kept walking.”

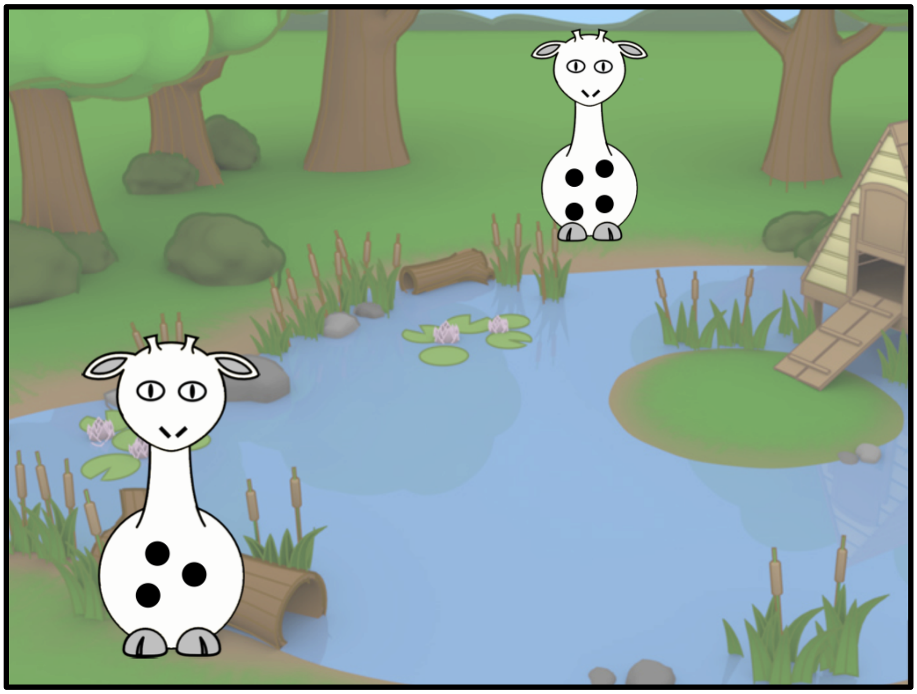

“Next, [Mr. Three/the giraffe with three spots] came to a pond of water. He saw another friend on the other side of the pond. This one [point] is not [Mr. Three/the giraffe with three spots], it’s his friend. Look at his friend’s tummy [point]! It has spots too, but it’s different. [Mr. Three/the giraffe with three spots] [point] said goodbye to his friend and kept walking.”

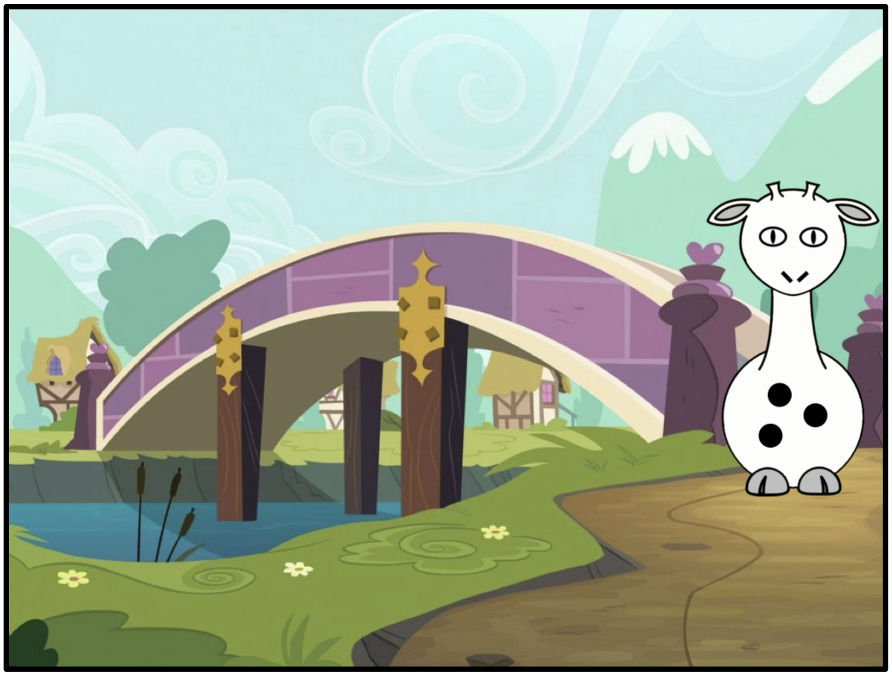

“[Mr. Three/the giraffe with three spots] walked over a bridge. Look at his tummy [point].”

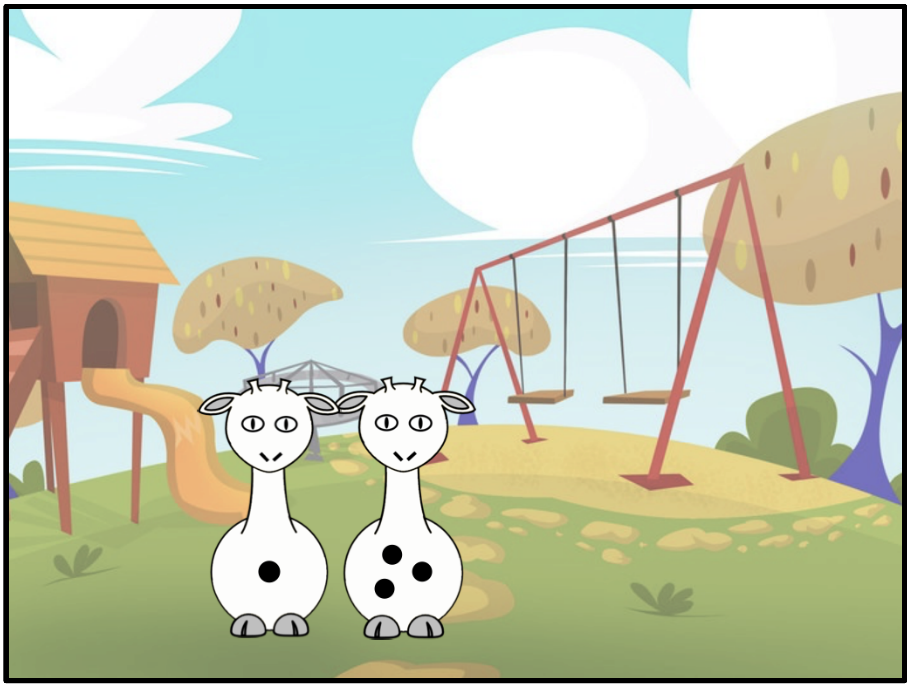

"[Mr. Three/the giraffe with three spots] came to a playground, where he met another friend. This one [point] is not [Mr. Three/the giraffe with three spots] -- it's his friend. Look at his friend's tummy [point]! It has spots too, but it's different. [Mr. Three/the giraffe with three spots] [point] said goodbye to his friend and kept walking."

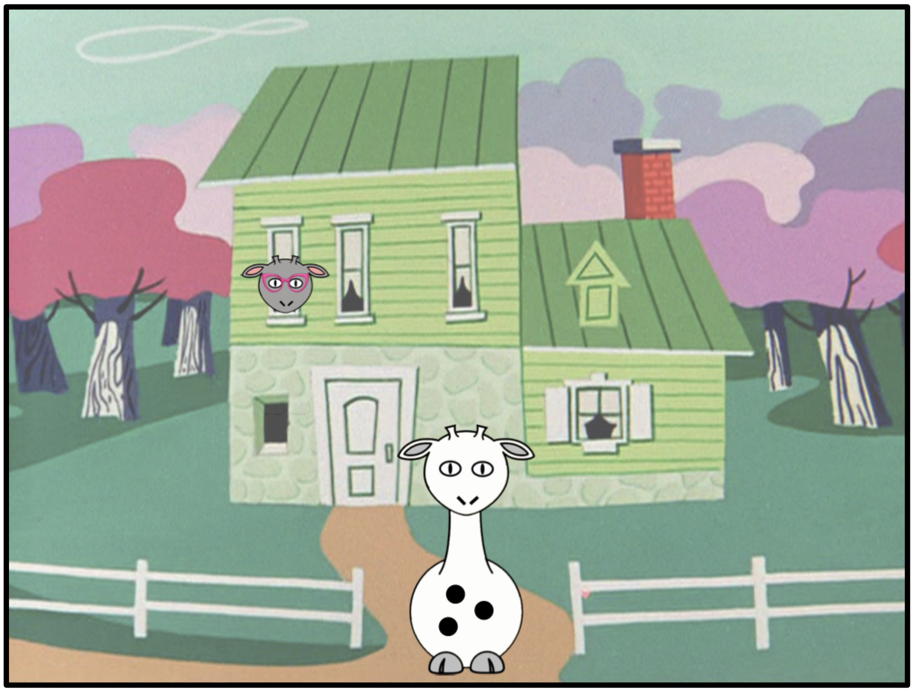

"Finally, [Mr. Three/the giraffe with three spots] [point] got to his grandma's house and knocked on the door. His grandma [point] came to the window and looked out at him."

1.3 Learning test trials:

“That’s the end of the story! Now you’re going to help me find [Mr. Three/the giraffe with three spots].”

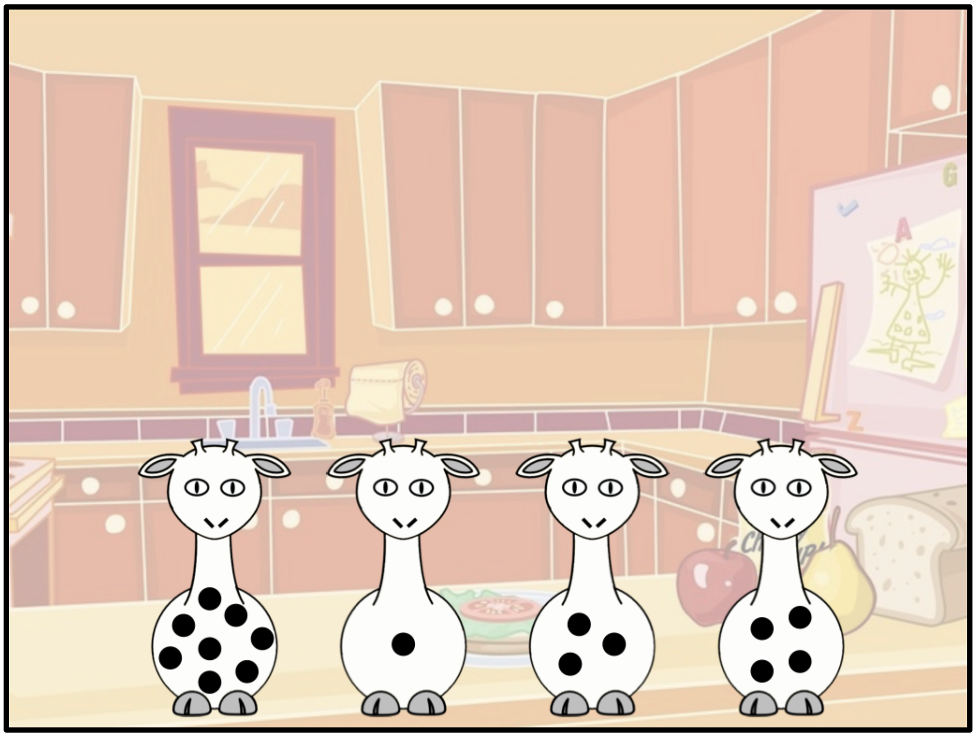

“Look, here’s [Mr. Three/the giraffe with three spots] and his friends in the kitchen. They all have spots on their tummies, but they’re all different. Can you point to [Mr. Three/the giraffe with three spots]?”

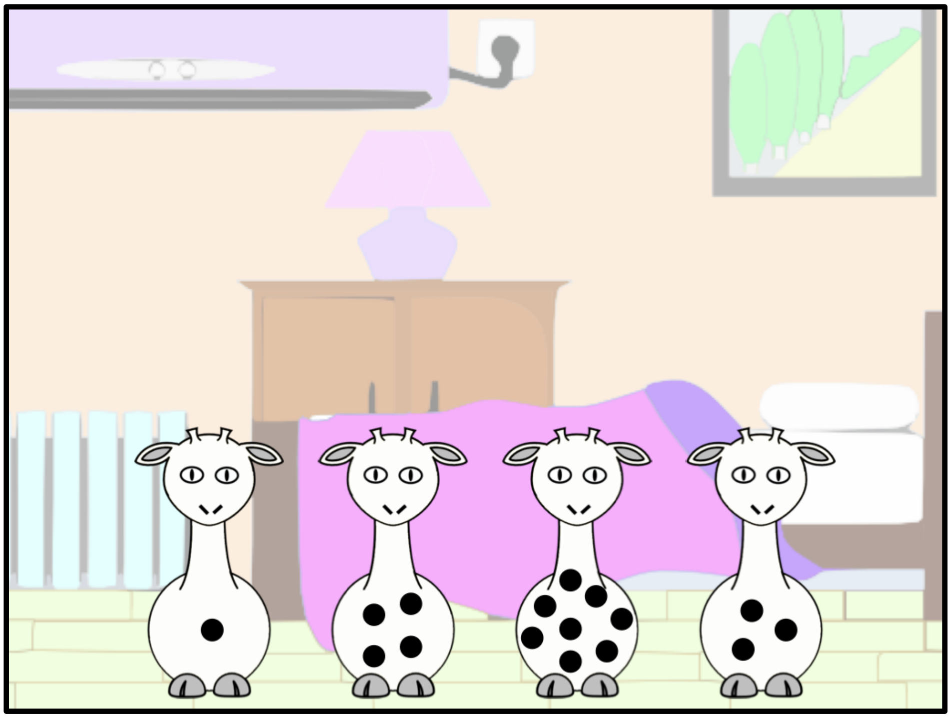

“Look, here’s [Mr. Three/the giraffe with three spots] and his friends in the bedroom. Can you point to [Mr. Three/the giraffe with three spots]?”

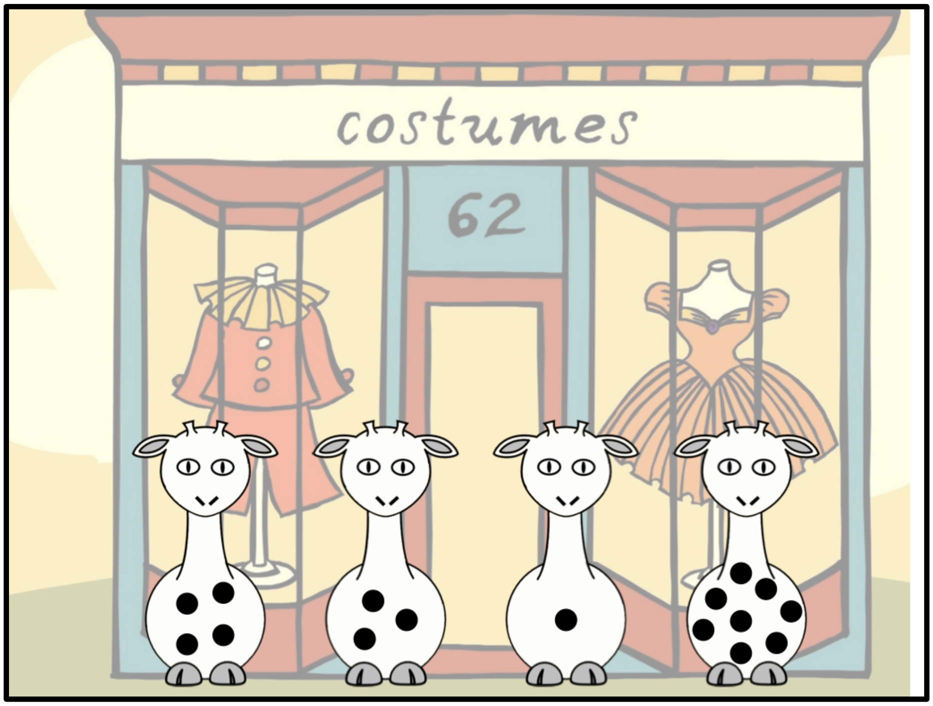

“Look, [Mr. Three/the giraffe with three spots]and his friends at the store. Can you point to [Mr. Three/the giraffe with three spots]?”

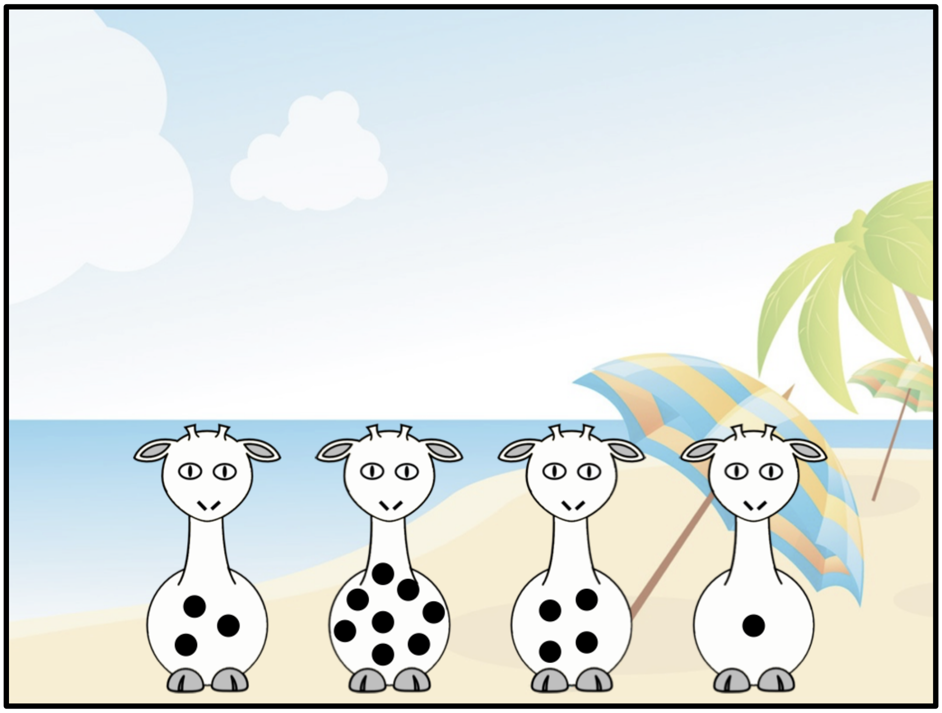

"Look, here's [Mr. Three/the giraffe with three spots]and his friends at the beach. Can you point to [Mr. Three/the giraffe with three spots]?"

1.4 Transfer test trials:

"Yay! Good job! Now, you're going to help me find [Mr. Three's grandma/the grandma with three spots]!"

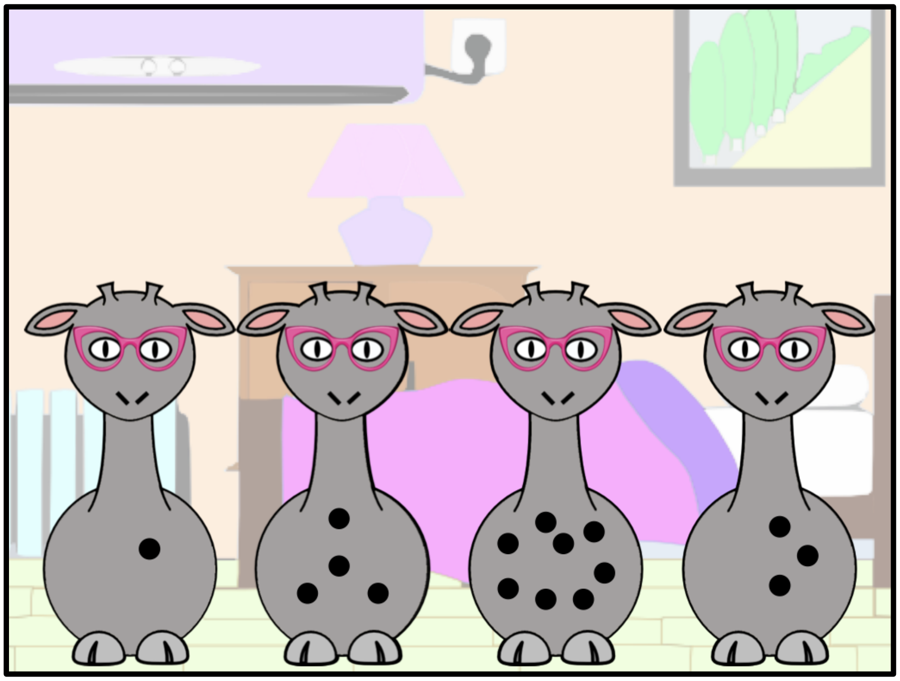

“Look, here’s grandma and her friends in the bedroom. They all have spots on their tummies, but they’re all different. Can you point to [Mr. Three’s grandma/the grandma with three spots]?”

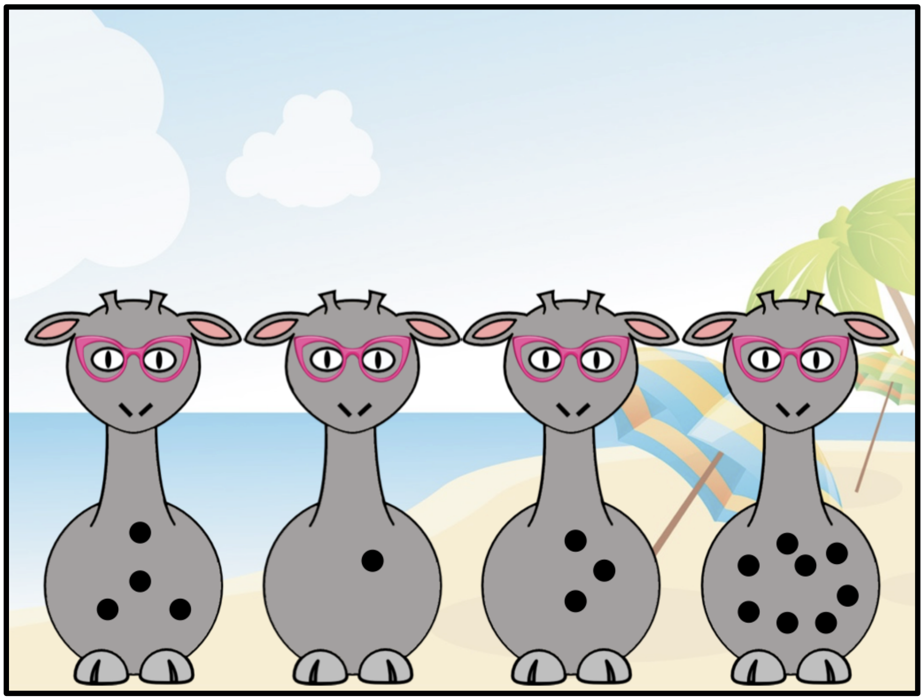

“Look, here’s grandma and her friends at the beach. Can you point to [Mr. Three’s grandma/the grandma with three spots]?”

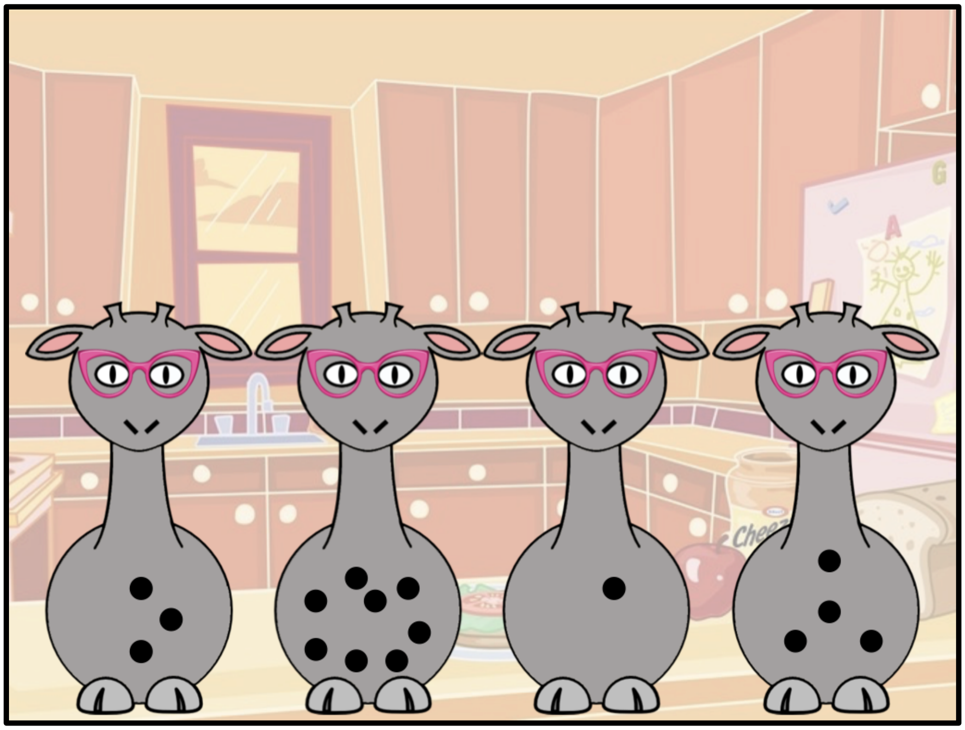

"Look, here's grandma and her friends in the kitchen. Can you point to [Mr. Three's grandma/the grandma with three spots]?"

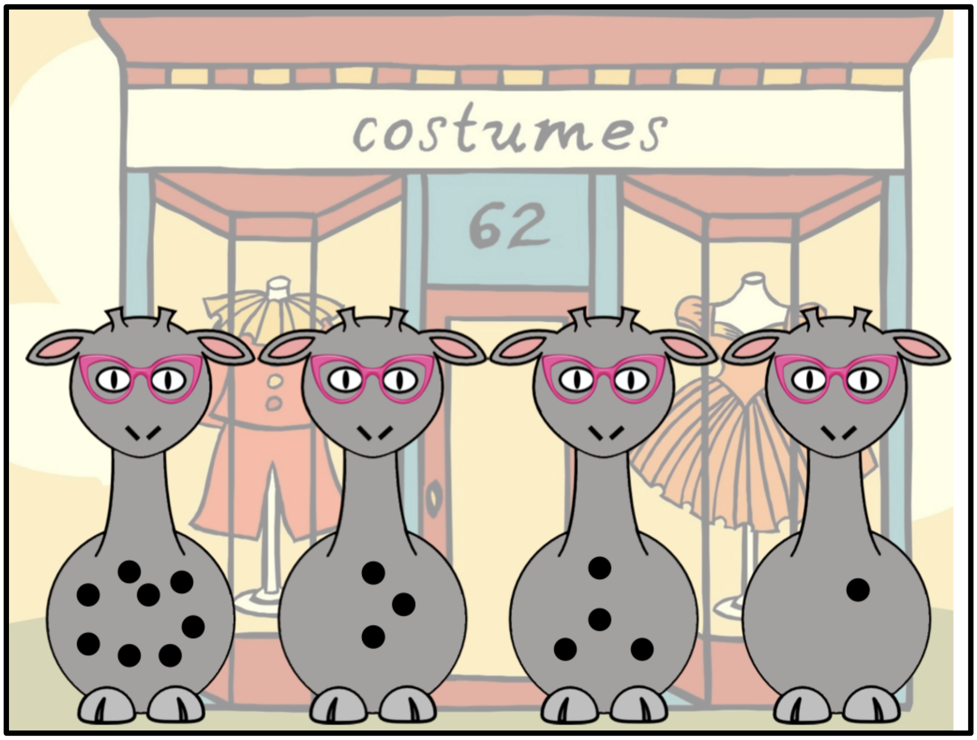

"Look, here's his grandma and her friends at the store. Can you point to [Mr. Three's grandma/the grandma with three spots]?"

2. Separate reports of two sub-experiments for Study 1

All data reported for Study 1 in the main text were combined across two experiments. The second experiment was a replication of the first, changing only the specificity of the recruitment criteria (to include only 1- and 2-knowers) and adding the "giraffe pretest" described in the Methods section of the main text. Critically, both the training and the test phases were identical in the two experiments. Here, we report the primary results of the original and replication experiments separately.

2.1 Experiment 1

2.1.1 Method

2.1.1.1 Participants

Participants in Experiment 1 were 90 2- and 3-year-olds (M_{age} = 2.8 years, range 2.0-3.9 years; 48 girls), including 35 non-knowers, 29 1-knowers and 26 2-knowers. Children were pseudo-randomly assigned to either the Proper Noun (n = 49, M_{age} = 2.8 years, range = 2.0-3.8 years) or the Adjective condition (n = 43, M_{age} = 2.9 years, range = 2.0-3.9 years). Children in the Proper Noun condition included 17 non-knowers, 18 1-knowers, and 12 2-knowers. Children in the Adjective condition included 18 non-knowers, 11 1-knowers, and 14 2-knowers.

Children were recruited from daycares and museums in the San Diego, CA (n = 75), Berkeley, CA, (n = 9), and Comox Valley, BC, (n = 6) areas. Only children who were not yet 3-knowers per the Give-a-Number pretest continued in the study. An additional 22 children were tested but excluded from analysis due to failure to complete all required trials (n = 15), speaking a primary language other than English (n = 2), parent interference (n = 4), parent-reported language delay (n =1).

2.1.1.2 Procedure

All methods were identical to those reported in the main text, with the exception that the giraffe pre-test was not conducted, and thus no participants were excluded due to passing it.

2.1.2 Results

2.1.2.1 Learning

To test whether Proper Noun training differed relative to Adjective training, we conducted a mixed-effects logistic regression predicting the likelihood of choosing the correct target, using Knower Level (non-knowers vs. 1-knowers vs. 2-knowers; an ordinal factor) and Condition (Proper Noun vs. Adjective) as predictors, and including an interaction term and a random effect of subjects. We found that the effect of Knower Level did not significantly improve the fit of this model, $\chi^2(1) = 5.2$, $p = 0.08$, nor did the effect of Condition, $\chi^2(1) = 1.3$, $p = 0.25$, and there was no evidence of an interaction, $\chi^2(1) = 4.5$, $p = 0.11$[1].

Exploratory *post-hoc* analyses indicated that knower-level and condition nevertheless impacted which groups of children performed better on the task than chance responding would predict. As shown in Figure S1A, in the Proper Noun condition, non-knowers correctly identified the target giraffe on 25% of trials (s.e.m = 8%), which was not significantly different from chance (i.e., 25% correct; Wilcoxon signed-rank test, V = 34, $p = 0.68$); 1-knowers correctly identified the target giraffe on 46% (s.e.m. = 9%) of trials, which was significantly greater than chance (V = 108.5, $p = 0.02$); and 2-knowers identified the target on 81% (s.e.m = 6%) of trials, also significantly higher than chance (V = 66, $p = 0.001$). Meanwhile, in the Adjective condition, non-knowers correctly identified the target of 36% of trials (s.e.m. = 8%), which was not significantly

[1] If non-knowers are removed from the sample in Experiment 1, the effect of knower-level reaches significance, $\chi^2(1) = 4.1$, $p = 0.04$, but the effect of condition does not, $\chi^2(1) = 3.6$, $p = 0.06$.

higher than chance (V = 41, p = 0.09); 1-knowers correctly identified the target on 30% of trials (s.e.m = 12%), which was also not different from chance (V = 28, p = 0.5); and 2-knowers did so on 59% of trials (s.e.m. = 12%), which was greater than chance (V = 60, p = 0.008).

2.1.2.2 Transfer

In order to assess whether children's knowledge of "three" extended beyond the specific exemplars they were trained on, but within the same basic category (giraffes), we examined performance on the Transfer trials, which involved a new set of giraffes with different spot configurations. A mixed-effects logistic regression with the same effects structure as that used on the Learning trials indicated that neither Condition ($\chi^2(1) = 0.50$, p = .48) nor Knower Level ($\chi^2(1) = 4.8$, p = .09), nor their interaction ($\chi^2(1) = 1.4$, p = .48) significantly improved the fit of the model predicting success on the Transfer trials relative to a null model.

As shown in Figure S1B, we found that, in the Proper Noun condition, non-knowers chose "Mr. Three's grandma" on 28% (s.e.m. = 5%) of trials, which was not different from chance (V = 33, p = .29), 1-knowers chose "Mr. Three's grandma" on 28% (s.e.m. = 6%) of trials, which was not different from chance (V = 36, p = .41), while 2-knowers chose the target grandmother on 54% (s.e.m. = 11%) of trials, which was significantly greater than chance (V = 41, p = 0.02). In the Adjective condition, non-knowers chose "the grandma with three spots" on 21% (s.e.m. = 7%) of trials, which was not different from chance (V = 28.5, p = 0.82); 1-knowers chose "the grandma with three spots" on 29% of trials (s.e.m. = 11%), which was not different from chance (V = 18.5, p = .5); and 2-knowers chose her on 43% (s.e.m. = 10%) of trials, which was only marginally greater than chance (V = 43, p = .06). To summarize, only 2-knowers showed any evidence of transfer, and this effect only reached statistical significance in the Proper Noun condition.

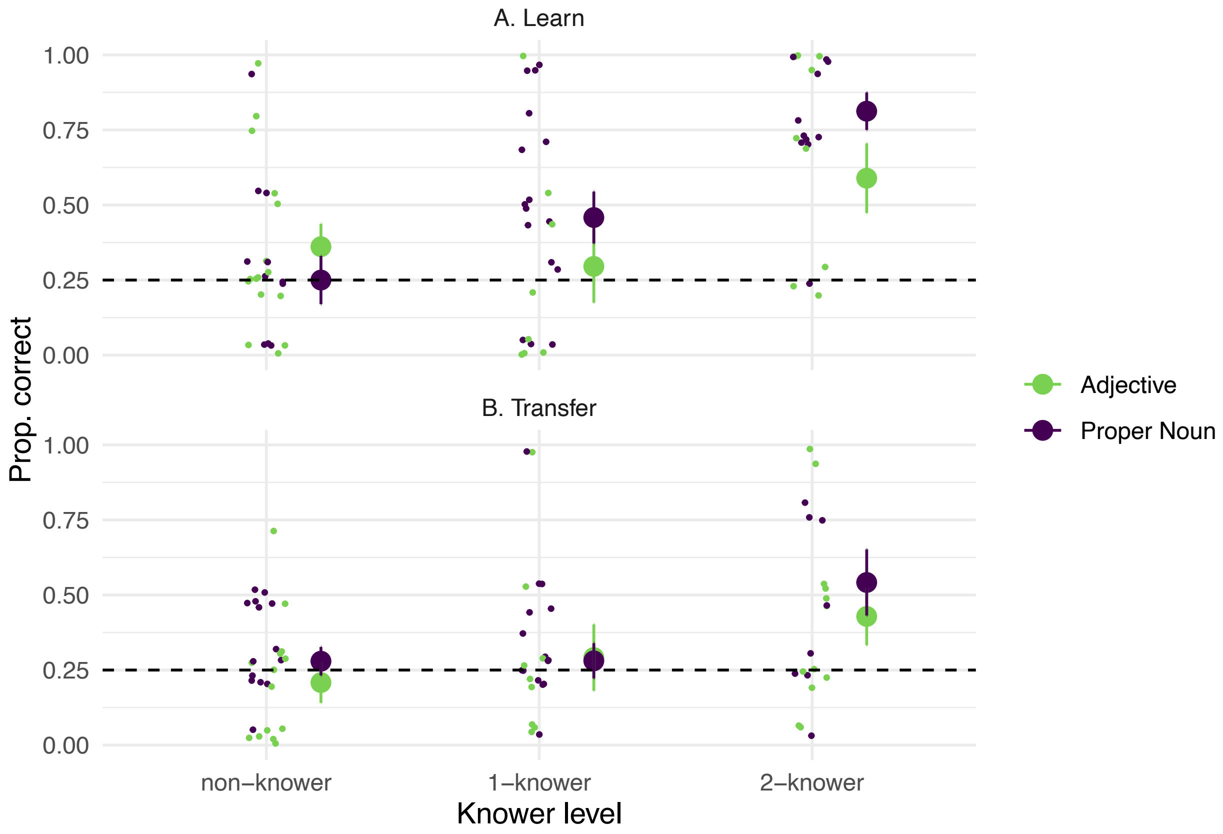

Figure S1. A. Proportion of trials in which children correctly identified either “Mr. Three” in the Proper Noun condition or “the giraffe with three spots” in the Adjective condition in Study 1, Experiment 1, after training. B. Proportion of trials in which children transferred their knowledge to correctly select either “Mr. Three’s grandma” in the Proper Noun condition or “the grandma with three spots” in the Adjective condition. Large dots = group means; Error bars = s.e.m; small dots = individual subject means with jitter; dashed line = chance performance.

2.1.3 Discussion

In Experiment 1, we did not detect significant effects of either condition or knower-level in our mixed-effects models of children’s performance on the Learning and Transfer trials. Nevertheless, we did see an interesting pattern on the Learning trials in which 1-knowers in the

Proper Noun condition were able to correctly identify the 3-spotted giraffe more often than chance responding would predict, but 1-knowers in the Adjective condition were not. Moreover, there was a large difference in the mean performance of 2-knowers in the two conditions: 2-knowers in the Proper Noun condition identified the target correctly on 81% of trials, while 2-knowers in the Adjective condition did so on only 59% of trials. These effects suggested to us that our models of the complete dataset, especially in the Learning trials, may have been underpowered and/or that learning effects may have been washed out by greater variability in number understanding among children classified as non-knowers (e.g., some may have been on the verge of learning "one," while others may have been many months away from this milestone). Consistent with this hypothesis, when we removed non-knowers from the sample, the effect of knower-level in our model of children's performance on the Learning trials became significant.

Because we did not recruit children by their specific knower-levels (i.e., any child who did not yet have a precise meaning for "three" was counted toward the target *n* for each condition), the sample sizes of subset-knowers (relative to non-knowers) were small, particularly in the Adjective condition (*n* = 11 1-knowers, 14 2-knowers). We therefore decided to conduct a replication study specifically recruiting 1- and 2-knowers and increasing the target *n* for each knower-level group to 40 children.

Additionally, although all children in Experiment 1 were pretested on the Give-a-Number task to ensure that they were not yet 3-knowers by Wynn's criteria, it was nevertheless possible that some of the children who successfully identified [Mr. Three/the giraffe with three spots] after training could have done so even without having experienced our training intervention. To address this potential concern in Experiment 2, we also added an additional "giraffe pretest" in which

children were asked to select the target from a "line-up" similar to the test trials *prior* to training, as described in the main text, and excluded all children who did so accurately from the sample.

2.2 Experiment 2

2.2.1 Method

2.2.1.1 Participants

A total of 89 children (M_{age} = 3.1 years, range = 2.1 - 4.0 years; 47 girls) participated in Experiment 2, including 47 1-knowers and 42 2-knowers. Children were pseudo-randomly assigned to either the Proper Noun (n = 44, M_{age} = 3.0 years, range = 2.2-3.8 years) or the Adjective condition (n = 45, M_{age} = 3.2 years, range = 2.1-4.0 years). Children in the Proper Noun condition included 23 1-knowers, and 21 2-knowers. Children in the Adjective condition included 24 1-knowers, and 21 2-knowers.

Children were recruited from daycares, preschools, and museums in the San Diego, CA, (n = 61) and Comox Valley, BC, (n = 28) areas. Only children who were classified as 1- or 2-knowers during the Give-a-Number pretest continued in the study. An additional 21 children were tested but excluded from all analyses due to failure to complete all required trials (n = 13) or to passing the "giraffe pretest" described in the Methods section of the main text (n = 8),

2.2.1.2 Procedure

Procedures were identical to those reported in the main text.

2.2.2 Results

2.2.2.1 Learning

To test whether Proper Noun training differed relative to Adjective training, we conducted a mixed-effects logistic regression predicting the likelihood of choosing the correct target, using Knower Level (1-knowers vs. 2-knowers; an ordinal factor) and Condition (Proper Noun vs.

Adjective) as predictors, and including an interaction term and a random effect of subjects. We found that the effect of Knower Level did not significantly improve the fit of this model, $\chi^2(1) = 1.7$, $p = .2$, nor did the effect of Condition, $\chi^2(1) = 2.3$, $p = .13$, and there was no evidence of an interaction, $\chi^2(1) = 0.07$, $p = .79$.

Although the model did not detect significant effects of knower-level and condition, in a *post-hoc* exploratory analysis, these factors impacted which groups of children performed better on the task than chance responding would predict. As shown in Figure S2A, in the Proper Noun condition, 1-knowers correctly identified the target giraffe on 44% (s.e.m. = 8%) of trials, which was significantly greater than chance (V = 97.5, $p = .02$); and 2-knowers identified the target on 62% (s.e.m = 9%) of trials, also significantly higher than chance (V = 155, $p = .001$). Meanwhile, in the Adjective condition, 1-knowers correctly identified the target on 34% of trials (s.e.m = 8%), which was not significantly different from chance (V = 99, $p = .14$); and 2-knowers did so on 49% of trials (s.e.m. = 9%), which was greater than chance (V = 116, $p = .006$).

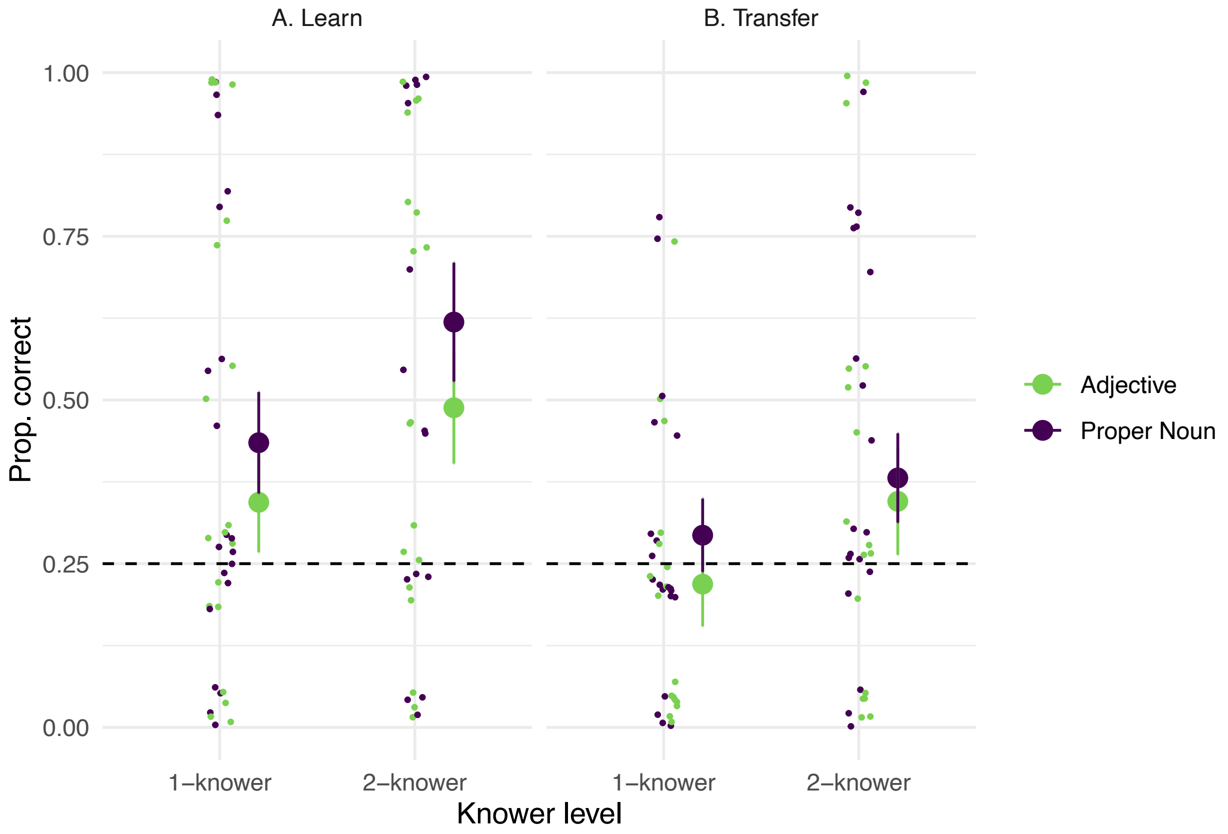

Figure S2. A. Proportion of trials in which children correctly identified either "Mr. Three" in the Proper Noun condition or "the giraffe with three spots" in the Adjective condition in Study 1, Experiment 2, after training. B. Proportion of trials in which children transferred their knowledge to correctly select either "Mr. Three's grandma" in the Proper Noun condition or "the grandma with three spots" in the Adjective condition. Large dots = group means; Error bars = s.e.m; small dots = individual subject means with jitter; dashed line = chance performance.

2.2.2.2 Transfer.

In order to assess whether children's knowledge of "three" extended beyond the specific exemplars they were trained on, we examined performance on the Transfer trials, which involved a new set of "grandmother" giraffes with different spot configurations. A mixed-effects logistic regression with the same effects structure as that used on the Learning trials indicated that neither

Condition ($\chi^2(1) = 1.1$, $p = .3$) nor Knower Level ($\chi^2(1) = 2.1$, $p = .14$), nor their interaction ($\chi^2(1) = 0.22$, $p = .64$) significantly improved the fit of the model predicting success on the Transfer trials relative to a null model.

As shown in Figure S2B, we found that, in the Proper Noun condition, 1-knowers chose "Mr. Three's grandma" on 29% (s.e.m. = 6%) of trials, which was not different from chance (V = 48, $p = .24$), while 2-knowers chose the target grandmother on 38% (s.e.m. = 7%) of trials, which was significantly greater than chance (V = 82.5, $p = .03$). In the Adjective condition, 1-knowers chose "the grandma with three spots" on 22% of trials (s.e.m.= 6%), which was not different from chance (V = 67, $p = .81$), while 2-knowers chose her on 35% (s.e.m. = 8%) of trials, which was also not different from chance (V = 84, $p = .2$). To summarize, only 2-knowers in the Proper Noun condition showed evidence of transfer.

2.2.3 Discussion

In Experiment 2, we successfully replicated Experiment 1 in a larger sample of 1- and 2-knowers. At the group level, the overall pattern of results for the two experiments was identical: We found that 2-knowers could be trained to identify "three" in the Learning trials with either Proper Noun or Adjective training, but that their mean performance level was higher after Proper Noun training. One-knowers, however, only performed above chance with Proper Noun training. Meanwhile, on the Transfer trials, only 2-knowers in the Proper Noun training group performed significantly better than chance responding would predict. However, we did not detect significant effects of condition or knower-level on either Learning or Transfer in our mixed-effects models of the Experiment 2 dataset.

In addition to producing the same pattern of results, Experiment 1 and Experiment 2 were nearly methodologically identical. The only differences between them were the addition of the

"giraffe pretest" (which screened out only 8 children) and the non-inclusion of non-knowers in Experiment 2. Therefore, we decided to test the hypothesis that the reason we did not find evidence in our modeling that knower-level and/or condition were significant predictors of children's performance in Experiment 1 or Experiment 2 alone was a lack of statistical power. In order to do this, we combined the data from 1-knowers and 2-knowers in both experiments (excluding the non-knowers from Experiment 1) and analyzed it as one larger, higher-powered dataset. The results of that analysis are reported in the main text.

3. Supplementary Tables

Table S1. Number of spots on giraffes chosen in error during learning and transfer trials in Study 1 (combined sample)

Condition	Children (N)	Errors (N)	Giraffe selected N (%)		
			1	4	8
Proper Noun	74	325	109 (34)	122 (38)	94 (29)
Adjective	70	352	67 (19)	101 (29)	184 (52)
Total	144	677	176 (26)	223 (33)	278 (41)

Table S2. Knower-levels before and after training on “three”

Condition	KL	N	Post-test knower level					
			0	1	2	3	4	CP
Proper Noun	1	36	5	24	6	1	0	0
	2	31	1	7	21	0	2	0
Adjective	1	34	6	24	4	0	0	0
	2	28	2	3	18	3	1	1

KL = knower-level prior to training.

4. Effects of Learning on Transfer

4.1 Mr. 3 (combined sample)

We were interested in how likely it was for children who had successfully learned the identity of the target giraffe on the Learning trials to also identify the correct target during the Transfer trials. For this analysis, we considered a child to have been successful at Learning if they correctly identified Mr. Three/the giraffe with three spots on at least 3 of 4 test trials (binomial test, p = .05). Of the 25 children in the Adjective condition who met this criterion for Learning (36% of the total sample in that condition), 40% (n = 10) also succeeded on at least 3 of 4 Transfer trials. Of the 35 children in the Proper Noun condition who met this criterion for Learning (47% of the sample in that condition), 34% (n = 12) also succeeded on the Transfer trials. The proportions of learners who transferred their knowledge were not significantly different in the

Adjective and Proper Noun conditions ($\chi^2(1) = 0.03$, $p = .9$). In contrast, only 2 children in the Adjective condition and 3 children in the Proper Noun condition who failed to meet the criterion for Learning nevertheless succeeded on the Transfer trials. In both conditions, the proportion of learners who succeeded on the Transfer trials was significantly higher than the proportion of non-learners who did so (both $\chi^2(1) > 5.3$, both $p < .05$).

4.2 Mr. Purple

In Study 2, we found that 9 children in the Proper Noun condition (35% of the sample in that condition) correctly identified Mr. Purple on at least 3 of 4 trials. Of these Learners, 44% (n = 4) also showed evidence of transfer, correctly identifying Mr. Purple's grandma on at least 3 trials. Meanwhile, in the Adjective condition, 8 children (27% of the sample in that condition) correctly identified the giraffe with purple spots on at least 3 trials, and, of those children, 25% (n = 2) also successfully selected the target on at least 3 of 4 trials during Transfer. In both conditions, only one child who did not show evidence of Learning nevertheless showed evidence of Transfer. Due to the very small number of children who succeeded at the criterion level of 3 transfer trials in this study, we were unable to determine whether the effects of learning on transfer differed significantly across the two conditions, or whether children who learned were statistically more likely to succeed at the transfer trials.
